# Elevated expression of WSB2 degrades p53 and activates the IGFBP3-AKT-mTOR-dependent pathway to drive hepatocellular carcinoma

**DOI:** 10.1038/s12276-023-01142-6

**Published:** 2024-01-04

**Authors:** Xun Li, Cheng-Cheng Zhang, Xiao-Tong Lin, Jie Zhang, Yu-Jun Zhang, Hong-Qiang Yu, Ze-Yu Liu, Yi Gong, Lei-Da Zhang, Chuan-Ming Xie

**Affiliations:** 1 Department of Hepatobiliary Surgery, Key Laboratory of Hepatobiliary and Pancreatic Surgery, Southwest Hospital, Third Military Medical University (Army Medical University), Chongqing, 400038 China; 2 Department of General Surgery, Guangxi Zhuang Autonomous Region Corps Hospital of Chinese People’s Armed Police Force, Nanning, 530003 China

**Keywords:** Ubiquitylation, Oncogenes, Oncogenes

## Abstract

Dysregulation of wild-type p53 turnover is a key cause of hepatocellular carcinoma (HCC), yet its mechanism remains poorly understood. Here, we report that WD repeat and SOCS box containing protein 2 (WSB2), an E3 ubiquitin ligase, is an independent adverse prognostic factor in HCC patients. WSB2 drives HCC tumorigenesis and lung metastasis in vitro and in vivo. Mechanistically, WSB2 is a new p53 destabilizer that promotes K48-linked p53 polyubiquitination at the Lys291 and Lys292 sites in HCC cells, leading to p53 proteasomal degradation. Degradation of p53 causes IGFBP3-dependent AKT/mTOR signaling activation. Furthermore, WSB2 was found to bind to the p53 tetramerization domain via its SOCS box domain. Targeting mTOR with everolimus, an oral drug, significantly blocked WSB2-triggered HCC tumorigenesis and metastasis in vivo. In clinical samples, high expression of WSB2 was associated with low wild-type p53 expression and high p-mTOR expression. These findings demonstrate that WSB2 is overexpressed and degrades wild-type p53 and then activates the IGFBP3-AKT/mTOR axis, leading to HCC tumorigenesis and lung metastasis, which indicates that targeting mTOR could be a new therapeutic strategy for HCC patients with high WSB2 expression and wild-type p53.

## WSB2: The hidden instigator of hepatocellular carcinoma and lung metastasis

Hepatocellular carcinoma (HCC, a common type of liver cancer) has a low survival rate due to incomplete understanding of its growth. In this research, scientists identified a protein named WSB2 that is overly produced in HCC and linked with poor patient results. The research involved 289 HCC patients and used different methods like gene sequencing, tissue analysis, and cell culture experiments. The scientists discovered that WSB2 interacts with a tumor suppressor protein (protein that prevents cancer growth) named p53, causing its breakdown and promoting cancer growth. This process was found to be independent of another protein, Mdm2, known to degrade p53. The research also showed that a medicine called everolimus, which inhibits a pathway activated by WSB2, could potentially be used to treat HCC. The findings provide new understanding into HCC growth and potential treatments.

This summary was initially drafted using artificial intelligence, then revised and fact-checked by the author.

## Introduction

Hepatocellular carcinoma (HCC), which represents approximately 90% of primary liver cancers, is estimated to be the sixth most common malignancy and the third leading cause of cancer-related death globally^1^. Despite improvements in therapeutic options for HCC, the mortality rate of HCC has increased, and the 5-year survival rate is below 20%^2^, because the mechanisms responsible for HCC development are still incompletely understood^3^. Hence, exploring the mechanisms of HCC tumorigenesis is urgently warranted.

p53 is the most intensively investigated tumor suppressor. Since its discovery in 1979, the important role of the p53 protein in tumorigenesis and development has been well established^4^. As the “guardian of the genome”, p53 triggers antagonistic cellular responses, including cell cycle arrest, apoptosis and senescence, in response to DNA damage or the aberrant activation of oncogenes^5^. p53 consists of an N-terminal transactivation domain, a core DNA-binding domain, and a C-terminal tetramerization domain. Genomic DNA sequencing of human cancers indicates that the p53 gene (TP53) is somatically mutated in only approximately 36% of HCC cases, and most HCC patients maintain low expression of wild-type p53^6^. However, the underlying mechanisms of the low expression of p53 in HCC remain incompletely elucidated. Increasing evidence has shown that loss of function of p53 caused by posttranslational modifications, such as ubiquitination, phosphorylation, methylation and acetylation, plays a critical role in HCC tumorigenesis and development^7^. Among them, ubiquitination is the most important and widely studied posttranslational modification pathway^8^. Ubiquitination can inhibit the functional activity of p53, promote the nuclear export of p53 and mediate its degradation through the proteasome pathway in the cytoplasm^9^.

Ubiquitination, as a major posttranslational modification, represents a key regulator of a variety of cellular processes, including proteasomal degradation, DNA repair, internalization and trafficking of receptor proteins^9^. E3 ubiquitin ligases, which act at the last step of a three-enzyme cascade including the E1 ubiquitin-activating enzymes and E2 ubiquitin-conjugating enzymes, play the core role in conferring specificity to the ubiquitination pathway through mediating the transfer of ubiquitin to the substrates^10^. E3 ubiquitin ligases mainly fall into three categories: really interesting new gene (RING)-finger type, homologous to E6-associated protein carboxyl terminus (HECT)-domain type, and RING-in-between-RING (RBR) type^11^. The two most well-characterized subfamilies of RING-finger type E3s are the SCF (Skp1-Cullin-F-box) and ECS (ElonginB/C-Cullin2/5-SOCS-box) ubiquitin ligase complexes^12^. Previous studies have revealed that a series of E3 ubiquitin ligases, such as mouse double minute 2 homolog (Mdm2), Tripartite motif 59 (TRIM59), COP1 and Pirh2, can degrade p53 and suppress the transcriptional activity of p53 through the ubiquitin‒proteasome pathway^13,14^. E3 ubiquitin ligases can modulate p53 activity, stability and subcellular functional localization^13,14^.

WD repeat and SOCS box containing protein (WSB) molecules harbor a variety of WD repeats in the N-terminus and a SOCS box at the C-terminus. According to the difference in their number of WD motifs, they are divided into two kinds, WSB1 and WSB2^15^. Because they contain two highly conserved motifs, WD repeats and the SOCS box, WSB molecules play important roles in RNA biosynthesis, cell cycle regulation, apoptosis and signal transduction^15^. Dysfunction of WSB1 is related to tumor initiation and progression^16^. Through its E3 ligase activity, WSB1 can promote the ubiquitination and proteasomal degradation of the von Hippel‒Lindau (pVHL) tumor suppressor and induce cancer invasion and metastasis^17^. Unlike WSB1, WSB2 is a little-studied protein. WSB2 levels were upregulated in patients with melanoma or breast cancer^18,19^, and there is no report about the effects of WSB2 in HCC. In addition, there is no report about the effects of WSB2’s E3 ligase activity in tumors, including HCC. Herein, we provide evidence showing that WSB2 could ubiquitinate and degrade wild-type p53 in hepatocellular carcinoma, followed by activation of mTOR signaling via the IGFBP3/AKT axis.

## Materials and methods

### Patients and tissue specimens

A total of 289 HCC patients who underwent surgical resection in the Department of Hepatobiliary Surgery, Southwest Hospital, Army Medical University (Chongqing, P. R. China) were enrolled in this study (Supplementary Fig. 1a). Data for 113 HCC patients hospitalized in Southwest Hospital from January 2010 to December 2010 were used to analyze 10-year survival rates. Data for 146 HCC patients hospitalized from January 2015 to December 2015 were used to analyze 5-year survival rates. We collected 30 new paired HCC tumors and adjacent normal liver tissues from January 2021 to March 2021 to analyze WSB2 mRNA and protein expression. All patients who met the following criteria were included: (a) diagnosis of primary HCC based on the criteria of the European Association for the Study of the Liver^20^ and (b) no prior treatment except surgical resection. Patients with other cancers (including intrahepatic cholangiocarcinoma or other liver cancers) or who died from other causes were excluded. This study was approved by the ethical review boards of Southwest Hospital. All participants were fully informed and signed related written consent forms. The patients were followed up once every 2 months after initial treatment in the first 2 years and then every 3 months thereafter. DFS/OS was defined as the interval between the surgery date and HCC recurrence/death.

### Cell culture

The hepatocellular carcinoma cell lines HepG2, SK-Hep1 and PLC/PRF/5 were purchased from ATCC. The cell lines L02, Huh7, Hep3B, SMMC7721, HCCLM3 and HEK-293T were obtained from the Chinese Academy of Sciences Shanghai Branch Cell Bank (Shanghai, China). All cell lines were cultured in Dulbecco’s modified Eagle’s medium DMEM (Gibco, USA) supplemented with 10% fetal bovine serum (FBS) (Gibco, USA) at 37 °C in an atmosphere of 5% CO2.

### Bioinformatics analysis

Clinical data for HCC patients were downloaded from TCGA (http://cancergenome.nih.gov). RNA-Seq data were expressed as FPKM (Fragments Per Kilobase Million). The independent HCC microarray datasets GSE84402, GSE14520, GSE36376 and GSE45436 were available on GEO (http://www.ncbi.nlm.nih.gov/geo). The different mRNA expression levels between cancer and adjacent normal tissues were collected from the UALCAN cancer database (http://ualcan.path.uab.edu/analysis.html). The relationships between the gene mRNA expression levels and OS or DFS were analyzed via GEPIA (http://gepia.cancer-pku.cn/) and UALCAN. The substrates of E3 ligase were predicted by UbiBrowser analysis (http://ubibrowser.bioit.cn/ubibrowser_v3).

### Quantitative reverse-transcription PCR (qRT‒PCR)

Total RNA was isolated from human tissues or cultured cells using RNAiso Plus reagent (TaKaRa, Japan) according to the manufacturer’s instructions. cDNAs were generated using the PrimeScript™ RT Reagent Kit with gDNA Eraser (Perfect Real Time) (TaKaRa, Japan). Then, the mRNA levels of the indicated genes were analyzed by qRT‒PCR using TB Green™ Premix Ex Taq™ II (TaKaRa, Japan) on the CFX96 TouchTM Real-time PCR Detection System. The results were calculated based on the threshold cycle (Ct), and the relative fold change was determined using the 2^-ΔΔct^ method. Each experiment was performed three times independently, and β-actin was used as an endogenous control. Primers are shown in Supplementary Table 9.

### Western blotting

Total protein was extracted from treated cells or tumor tissues in RIPA lysis buffer and separated by 8% or 10% sodium dodecyl sulfate‒polyacrylamide gel electrophoresis (SDS‒PAGE) (Beyotime, China) before being transferred onto NC membranes (GE Healthcare, UK). The membranes were blocked in 5% skim milk for 1 hour at room temperature and then incubated with primary antibody overnight at 4 °C. Subsequently, the membranes were incubated with secondary antibody for 1 hour at room temperature. The immunoreactive bands were detected using Clarity TM Western ECL substrate (Bio-Rad, USA) and a Bio-Rad GelDoc system (Bio-Rad, USA). The primary antibodies and dilutions used are listed in Supplementary Table 11.

### Immunohistochemistry (IHC) staining assay

Following the manufacturer’s protocol, an immunohistochemistry kit (ZSGB-BIO, China) was used for immunohistochemical (IHC) staining. Briefly, after being dewaxed, rehydrated and antigen retrieved, the sections were inhibited by 3% hydrogen peroxide for 1 hour. Primary antibodies were incubated with the tissue sections at 4 °C overnight, and secondary antibody was applied for 1 hour at room temperature the next day. Slides were developed with DAB and counterstained with hematoxylin. The staining was evaluated by different specialized pathologists without any knowledge of the patient characteristics. The staining intensity was determined using the Spot Denso function of AlphaEaseFC software. The primary antibodies and dilutions used are listed in Supplementary Table 11.

A semiquantitative scoring system was used to determine the WSB2 protein levels. (a) The percentage of positively stained cells was scored as follows: “0” (<5%), “1” (5–25%), “2” (26–50%), “3” (51–75%) or “4” (>75%). (b) Intensity was scored as follows: “0” (negative staining), “1” (light yellow), “2” (brownish yellow) or “3” (brown). (c) The total Score= the percentage score × the intensity score. They were divided into negative (−, score 0), weakly positive (+, score 1–4), moderately positive (++, score 5-8) or strongly positive (+++, score 9-12). For the purpose of statistical analysis, patients were classified into a high-expression group (++, +++) and a low-expression group (−, +).

### Generation of plasmids, siRNA and lentivirus

The plasmids of the human WSB2 gene tagged with 3xFlag, human p53 tagged with HA and human IGFBP3 tagged with HA were constructed by GeneChem (China, Shanghai). siRNAs were synthesized by GenePharma (China, Shanghai). The deletion mutant plasmids of WSB2 and p53 were constructed by Tsingke Biotechnology (China, Beijing). The mutant plasmids that contained lysine-to-arginine substitutions of p53 were also constructed by Tsingke Biotechnology (China, Beijing). WSB2-overexpressing and negative control lentiviruses were purchased from GeneChem (China, Shanghai). The sequences are shown in Supplementary Table 10.

### Cell proliferation and colony formation assays

For the cell proliferation assays, 3000~5000 HCC cells were seeded into a 96-well plate for 24 hours and then transfected with the indicated plasmids or siRNAs using Lipofectamine 2000 (Invitrogen, USA). At the indicated time points, the viability of HCC cells was determined by Cell Counting Kit 8 (CCK-8, Dojindo, Japan) and measured at 450 nm using an enzyme-linked immunosorbent assay plate reader (Thermo Fisher, USA).

For colony formation assays, 800-1000 treated HCC cells were plated in 6-well plates and cultured for 2 weeks. Then, the colonies were fixed with 4% paraformaldehyde and stained with 0.1% crystal violet. The numbers of colonies with ≥50 cells were counted. Triplicates were performed in each group.

### Cell migration and invasion assays

Cell migration and invasion were evaluated by transwell assay. Chambers with an 8 μm pore size (Corning, USA) were used as previously described. Briefly, 1 × 10^4^ cells in 200 μl serum-free medium were added to the upper chamber with (for cell invasion) or without (for cell migration) 50 μl BD Matrigel mixture (diluted at 1:8 with DMEM), and 600 μl DMEM containing 20% FBS was added to the lower chamber. Forty-eight hours later, cells on the bottom surface of the chamber were fixed in 4% formaldehyde and stained with 0.1% crystal violet. The migrated cells were observed and photographed using an EVOS XL Core Imaging System (Thermo Fisher, USA). All experiments were performed in triplicate.

### Cell cycle assay

The cell cycle was analyzed using a cell cycle kit (Beyotime, #C1052) according to the manufacturer instructions. Briefly, after transfection with siRNA targeting WSB2 for 48 hours, the cells were fixed with 70% ethanol at 4 °C for 2 hours and then stained with propidium iodide (PI) containing RNase A at room temperature for 15–30 min in the dark. The cells were analyzed by flow cytometry (BD FACS Calibur, BD Biosciences, USA) using excitation wavelengths at 480 nm and emission wavelengths at 580 nm. Triplicate measurements were performed for cell cycle distribution analysis.

### Apoptosis assay

The Annexin V-FITC/7-AAD apoptosis detection kit (BestBio, #BB-4102) was used according to the manufacturer instructions. HCC cells (1 × 10^6^) were resuspended in 1× Annexin V binding buffer and stained with Annexin V-FITC/7-AAD dye solution. Cell apoptosis was detected using a flow cytometer (BD FACS Calibur, BD Biosciences, USA). Samples were prepared and assessed in triplicate for each group.

### Coimmunoprecipitation (Co-IP) and mass spectrometry assay

Co-IP assays were performed as previously described^21^. In brief, cells were transfected with the indicated plasmids for 48 hours. Then, the cells were collected and lysed in immunoprecipitation lysis buffer (Beyotime, China). Ninety percent of the cell lysates were added to the indicated primary antibody and 50 µl Protein A/G PLUS-Agarose (Santa Cruz, CA, USA). After incubation at 4 °C on a rotating device overnight, the immunoprecipitates were washed four times with immunoprecipitation buffer. The immunocomplexes were collected and used in a Western blotting assay with the indicated antibody. The immunocomplexes were subsequently subjected to mass spectrometry analysis by Shanghai Applied Protein Technology Co. Ltd. (Shanghai, China).

### In vivo ubiquitination assay

In vivo ubiquitination assays were performed as previously described^21^. Briefly, cells were transfected with the indicated plasmids. After 48 hours of transfection, the cells were treated with 10 μM MG132 for 5 hours before harvest. The cells were lysed in a series of ubiquitination buffers and Ni-NTA beads (Qiagen, Valencia, CA). Then, the beads were washed with a series of ubiquitination buffers. The elution was analyzed by Western blotting with the indicated antibodies.

### Protein half-life assays

After cells stably expressed the indicated vectors in 6-well plates, 100 μg/ml cycloheximide (CHX, Selleck, USA) was added to the cell medium. At the indicated time points, cells were harvested, lysed and subjected to Western blotting analysis. The band intensity was quantified by ImageJ software.

### Immunofluorescence staining assay

The localization of WSB2 and p53 was investigated by immunofluorescence. Briefly, cells were cultured on confocal dishes and transfected with the indicated plasmids (Beyotime, China). Forty-eight hours after transfection, the cells were subsequently fixed with 4% paraformaldehyde and permeabilized with 0.3% Triton X-100. After blocking, the cells were incubated with primary antibodies at 4 °C overnight and secondary antibodies with fluorophore labels at room temperature for 1 hour the next day. Hoechst 33342 was used to stain the nuclei of cells. Cell images were captured using a Zeiss LSM880 microscope (Zeiss, German). The primary antibodies and dilutions used are listed in Supplementary Table 11.

### The CRISPR/Cas9 system

We used a dual gRNA approach to knock out the full-length sequence of Mdm2 with the CRISPR/Cas9 system (Ubigene, Guangzhou, China). Briefly, dual gRNA and donor vectors were cotransfected into 293 T cells. One week later, the transfected cells were subjected to puromycin (1 μg/ml) selection, sorted into 96-well plates to form an independent clone, and then expanded into 24-well plates. Potential clones were further verified by Tsingke Biotechnology (China, Beijing).

### p53 mutation screening

The highly conserved exons 5 to 8 of the p53 gene were screened according to previous research^22^. The primers used in PCR are listed in Supplementary Table 9. HCC tissue samples were screened for p53 mutations by PCR followed by Sanger sequencing (ABI Prism 3730).

### Animal experiments

Male BALB/c athymic nude mice, aged 6 weeks, were purchased from Chengdu Gembio Bioscience Co. Ltd. (Sichuan, China) and were fed under specific pathogen-free conditions. The whole procedure was performed following the “Guide for the Care and Use of Laboratory Animals” (NIH publication 86-23, revised 1985) and was approved by the Committee on the Ethics of Animal Experiments of Southwest Hospital.

To establish the subcutaneous xenograft tumor model, mice were randomized into two groups, and 1 × 10^6^ tumor cells in a 1:1 (v/v) mixed with Matrigel were injected into the flanks of each mouse. Tumor volume was measured every 5 days. One month later, tumor weight was measured after the mice were sacrificed. Tumor volume was calculated with the following formula: volume = π/6 × length × width^2^.

To establish the orthotopic xenograft tumor model, subcutaneous tumors from WSB2-overexpressing HepG2 cells and HepG2-WT cell-bearing mice were cut into small pieces (1 mm^3^). After the mice were anesthetized, the rectus abdominis muscle was cut, and the upper peritoneum was opened to expose the liver tissue. A small incision was made in the left lateral lobe of the liver, and small tumor tissue pieces were excised before the abdomen was closed (5/6 mice per group). Tumors were harvested from all mice 6 weeks after transplantation. Tumor volumes and tumor weights were measured as described above. Drug treatments were initiated on Day 5 after tumor implantation. Tumor-bearing mice were intravenously injected with everolimus (5 mg/kg, i.p., twice per week) for 5 weeks. All mice were harvested 6 weeks after transplantation.

To establish an experimental lung metastasis model, 1 × 10^6^ tumor cells from different treatment groups were injected into the tail veins of mice (6 mice per group) for 7 weeks. All mice were harvested 8 weeks after transplantation. All tumors were collected and subjected to H&E staining and histological evaluation (paraffin section).

### Statistical analyses

The results are expressed as the mean ± standard error of the mean (SEM). SPSS 25.0 (SPSS, Chicago, IL, USA), GraphPad Prism 8.0 (GraphPad, San Diego, CA) and R version 4.1.0 (http://www.r-project.org/) were used in this study to analyze the data. The Pearson χ2 test or Fisher’s test was performed to compare categorical variables. Comparisons between 2 continuous variable groups were performed with a 2-tailed unpaired t test. When more than 2 groups were compared, one-way analysis of variance (ANOVA) with Tukey’s post hoc test was used when data passed the Shapiro‒Wilk normality test. Survival curves were generated by the Kaplan‒Meier method and compared using the log-rank test. Univariate and multivariate Cox regression analyses were performed to analyze the parameters associated with the overall survival (OS) and disease-free survival (DFS) of HCC patients. Nomograms can provide a more individualized and visualized manner to predict prognostic information in patients. A higher total score based on the sum of the assigned number of points for each factor in the nomograms means a worse prognosis. Here, a nomogram and its calibration curve were developed using the package rms in R version 4.1.0. The concordance index (C-index) was used to examine the performance of the nomogram. *p* < 0.05 was considered to indicate statistical significance.

## Results

### WSB2 is a potential p53 destabilizer, and overexpression of WSB2 is associated with a poor prognosis in HCC

According to the UbiBrowser database (http://ubibrowser.bioit.cn/ubibrowser_v3/Home/Result/index/name/P04637/module/Strict/proteinType/noE3), there are 46 known and 334 predicted E3 ubiquitin ligases that may ubiquitinate p53 (Supplementary Fig. 1b; Supplementary Table 1). Then, we analyzed the effect of the expression levels of the top 200 predicted E3 ligases on survival in the HCC (LIHC) cohort of The Cancer Genome Atlas (TCGA) using the UALCAN cancer database (http://ualcan.path.uab.edu/analysis.html); the levels of 71 E3 ligases were associated with prognosis (*p* < 0.05). Furthermore, 19 E3 ligases may be the key destabilizers of p53, with *p* values less than 0.0001 (Supplementary Fig. 1c–u; Supplementary Table 1). Among them, 13 E3 ligases were reported as p53 destabilizers, and 6 ligases have unknown functions (Supplementary Fig. 1p–u). We noticed that the top predicted ubiquitination substrate for WDR77 and WSB2 was p53 (Supplementary Fig. 2). WDR77 was reported to negatively regulate HBV replication in the liver^23^, suggesting that WDR77 is negatively associated with liver disease. It is possible that WSB2 controls p53 stability and plays a key role in regulating HCC.

**Fig. 1:**
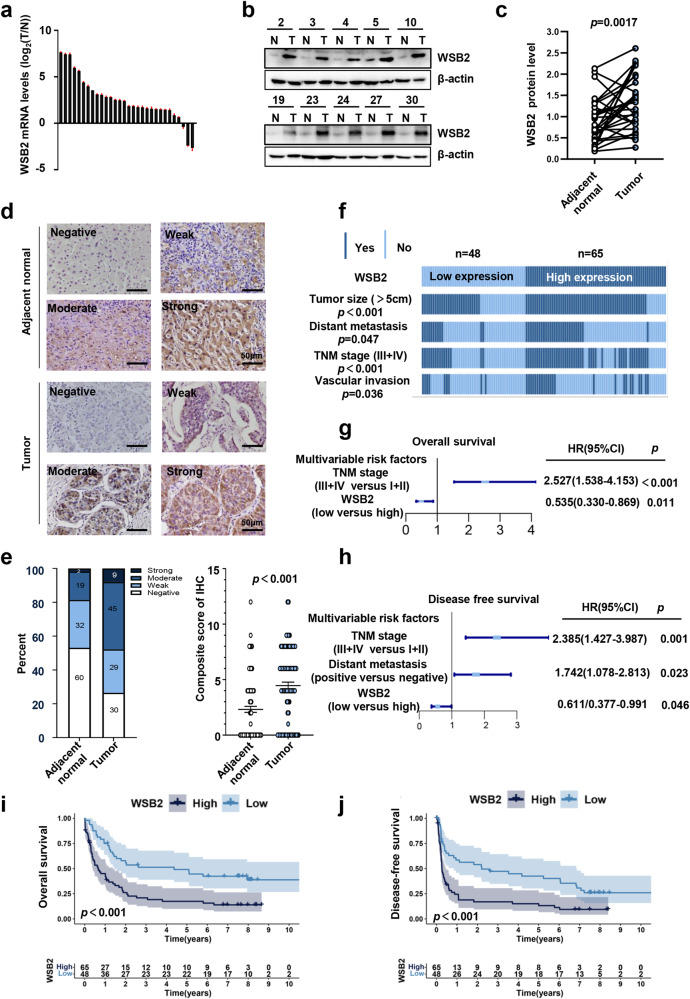
High WSB2 expression is correlated with a poor prognosis in HCC patients. **a** The relative WSB2 mRNA levels in 30 paired HCC and adjacent normal tissues were analyzed by qPCR. **b**, **c** WSB2 protein expression in 30 paired HCC and adjacent normal tissues**. d** Representative IHC staining images showing the expression level of WSB2 in HCC and adjacent normal tissues. Scale bar, 50 μm, *n* = 113. **e** The different expression levels of WSB2 in HCC tumors and adjacent normal liver tissues. *n* = 113. **f** High WSB2 expression is correlated with aggressive tumor phenotypes. **g** Multivariate analyses showing the risk factors associated with OS (overall survival) in the IHC cohort. *n* = 113. **h** Multivariate analyses showing the risk factors associated with DFS (disease-free survival) in the IHC hospital cohort. *n* = 113. **i**, **j** WSB2 was positively associated with poor OS (**i**) or DFS (**j**) in HCC patients. *n* = 113. Statistical significance was assessed by paired 2-tailed t test (**c**), Wilcoxon 2-tailed t test (**e**), χ2 test (**f**), univariate and multivariate Cox regression (**g**, **h**) or log-rank t test (**i**, **j**).

To further investigate the effect of WSB2 in HCC tissues, WSB2 expression levels were analyzed in 30 paired HCC tumors and adjacent normal liver tissues by qPCR and Western blotting. Consistent with WSB2 mRNA expression in the liver cancer (LIHC) cohort in the UALCAN database (Supplementary Fig. 3a, b) and Gene Expression Omnibus (GEO) database (Supplementary Fig. 3c–f), WSB2 expression was markedly increased in HCC tumor tissues compared to the matching adjacent normal liver tissues at both the mRNA level (Fig. 1a) and protein level (Fig. 1b, c). To further confirm these results, we analyzed WSB2 expression in another 113 paired HCC tissues and corresponding adjacent normal liver tissues by IHC staining. A total of 39.8% (45/113) of HCC tissue samples had a moderate level of WSB2 expression and 8.0% (9/113) had a high level of expression, whereas 16.8% (19/113) of normal tissue samples had a moderate level of WSB2 expression and 1.8% (2/113) had a high level of expression (Fig. 1d, e). The patients were then divided into WSB2 high (moderate and strong signal) and low (negative and weak signal) expression groups. High WSB2 expression was positively correlated with large tumor size (*p* < 0.001), vascular invasion (*p* = 0.036), distant metastasis (*p* = 0.047) and advanced TNM stage (*p* < 0.001) (Fig. 1f; Supplementary Table 2). According to univariate and multivariate regression analyses, WSB2 and TNM stage were strongly associated with the overall survival (OS) and recurrence in HCC patients (Fig. 1g–j; Supplementary Tables 3, 4), indicating that WSB2 may be a potential independent risk factor in HCC. Consistently, WSB2 expression at the mRNA level was positively associated with poor survival in HCC patients in the TCGA HCC cohort (http://gepia.cancer-pku.cn/, Supplementary Fig. 3g, h). Nomograms showed that WSB2, as an independent prognostic factor, could predict the survival status of patients after resection of HCC (Supplementary Fig. 3i–l). Taken together, these results indicate that WSB2 may be associated with liver tumorigenesis, metastasis, and OS in HCC patients.

Next, we further investigated the status of the TP53 gene in 113 HCC tissue samples: 79 samples were TP53 wild-type, and 34 samples were TP53 mutated. Interestingly, we found that the poor prognosis of HCC patients with high expression of WSB2 depended on TP53 gene status. WSB2 was positively associated with large tumor size (*p* = 0.001), vascular invasion (*p* = 0.002), distant metastasis (*p* = 0.016) and advanced TNM stage (*p* < 0.001) in HCC patients with wild-type TP53 but not mutant TP53 (Supplementary Fig. 4a, b; Supplementary Tables 5, 6). In line with this finding, WSB2 was negatively correlated with OS and DFS in HCC patients with wild-type TP53 but not in those with mutant TP53 (Supplementary Fig. 4c–f).

### WSB2 promotes HCC tumorigenesis and lung metastasis in vivo

**Fig. 2:**
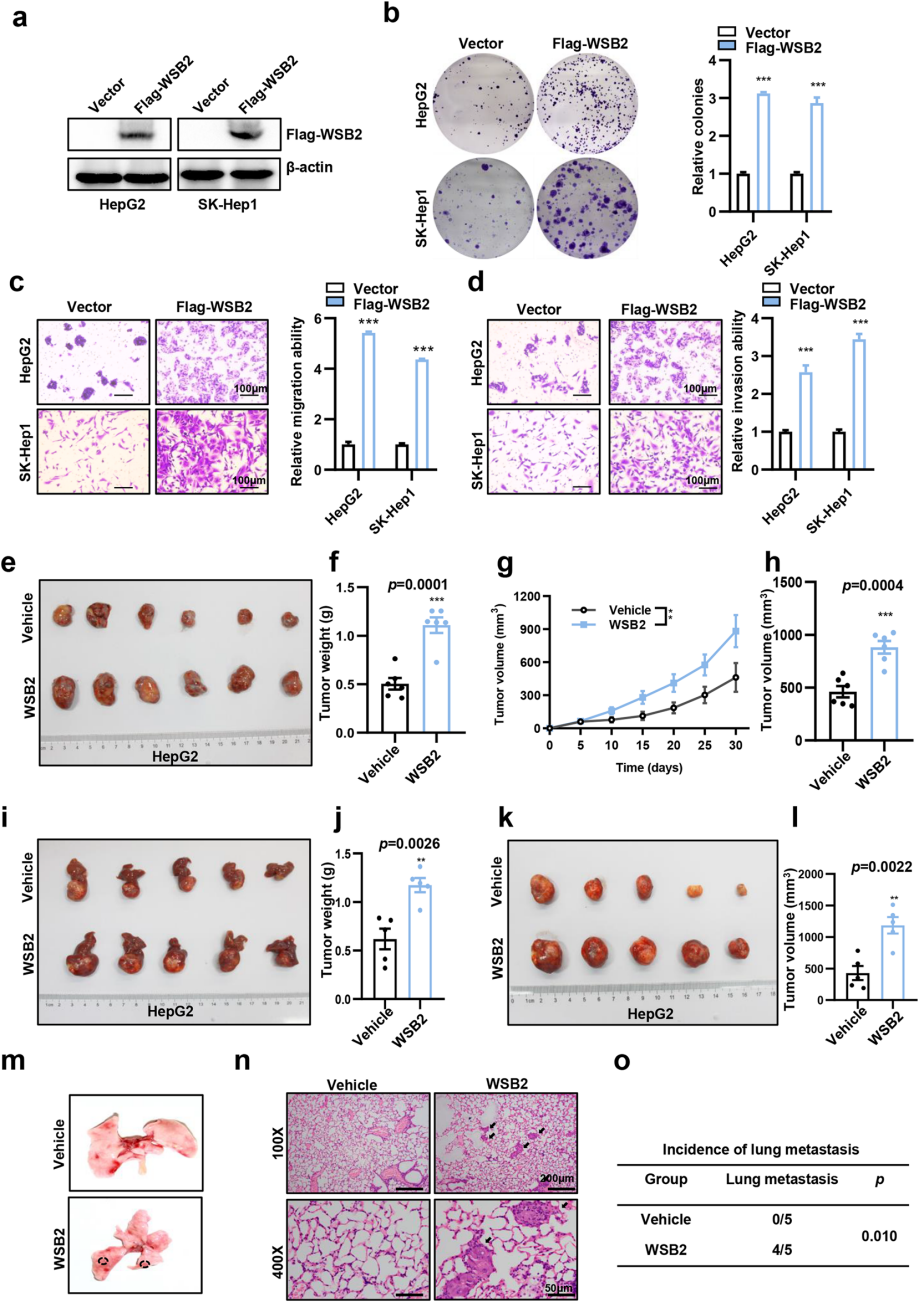
WSB2 promotes HCC tumorigenesis and lung metastasis in vitro and in vivo. **a** The efficiency of WSB2 overexpression in the indicated HCCs was detected by Western blotting. **b** Representative images and quantification of colony number are shown in cells transfected with Flag-WSB2 plasmids. *n* = 3. **c** Representative images and quantification of cell migration of HepG2 and SK-Hep1 cells after Flag-WSB2 overexpression. *n* = 3. Scale bar, 100 μm. **d** Representative images and quantification of cell invasion of HepG2 and SK-Hep1 cells after Flag-WSB2 overexpression. *n* = 3. Scale bar, 100 μm. **e**–**h** A macroscopic view of a subcutaneous tumor in mice injected with WSB2-overexpressing HepG2 cells (*n* = 6 mice/group). Tumor weights were determined, and tumor size was determined. **i**–**l** A macroscopic view of orthotopic tumors in the livers of mice injected with WSB2-overexpressing HepG2 cells (*n* = 5 mice/group). Tumors were imaged, and tumor weight was determined. **m** Representative macroscopic images showing lung metastatic nodules in the WSB2-overexpressing group (indicated by black circles). **n** H&E staining images showing lung metastatic nodules (indicated by black arrows). **o** The incidence of lung metastases in the WSB2-overexpressing group and control group was quantified. *n* = 5 mice/group. Data are shown as the means ± SEMs. Statistical significance was assessed by Student’s two-tailed t-test (**b**–**d**, **f**, **h**, **j**, **l**), two-way analysis of variance (**g**) or χ2 test (**o**). **p < 0.01, ***p < 0.001.

WSB2 was highly expressed in most HCC cell lines compared to the normal human hepatic cell line L02 (Supplementary Fig. 5a, b). To test the effect of WSB2 on HCC cell proliferation and metastasis, we knocked down or overexpressed WSB2 in HepG2 and SK-Hep1 cells that harbored wild-type p53. We found that knockdown of WSB2 significantly inhibited cell proliferation, colony formation, cell migration and invasion in TP53 wild-type HCCs, whereas overexpression of WSB2 promoted these actions (Fig. 2a–d, Supplementary Fig. 5c–h). Surprisingly, knockdown or overexpression of WSB2 did not have any effect on cell proliferation, colony formation or metastasis in TP53 mutant-type cells (PLC/PRF/5 and Hep3B) (Supplementary Fig. 6). These findings demonstrate that the oncogenic role of WSB2 in HCC depends on p53 status.

Next, we examined the effect of WSB2 on hepatocellular carcinogenesis in vivo. WSB2-overexpressing HepG2 cells were injected subcutaneously into nude mice. The subcutaneous tumors in WSB2-overexpressing nude mice were larger and heavier than those in the control group (Fig. 2e–h). Furthermore, subcutaneous xenograft tumors were inoculated into the liver in an orthotopic xenograft tumor model. Overexpression of WSB2 significantly increased the orthotopic liver tumor volume and tumor weight compared to controls (Fig. 2i–l). The incidence rate of lung metastases in the WSB2-overexpressing group was significantly higher than that in the control group (Fig. 2m–o). All these data indicated that WSB2 promoted HCC tumorigenesis and metastasis in vitro and in vivo.

### WSB2 interacts with p53 at the tetramerization domain and shortens the half-life of p53

**Fig. 3:**
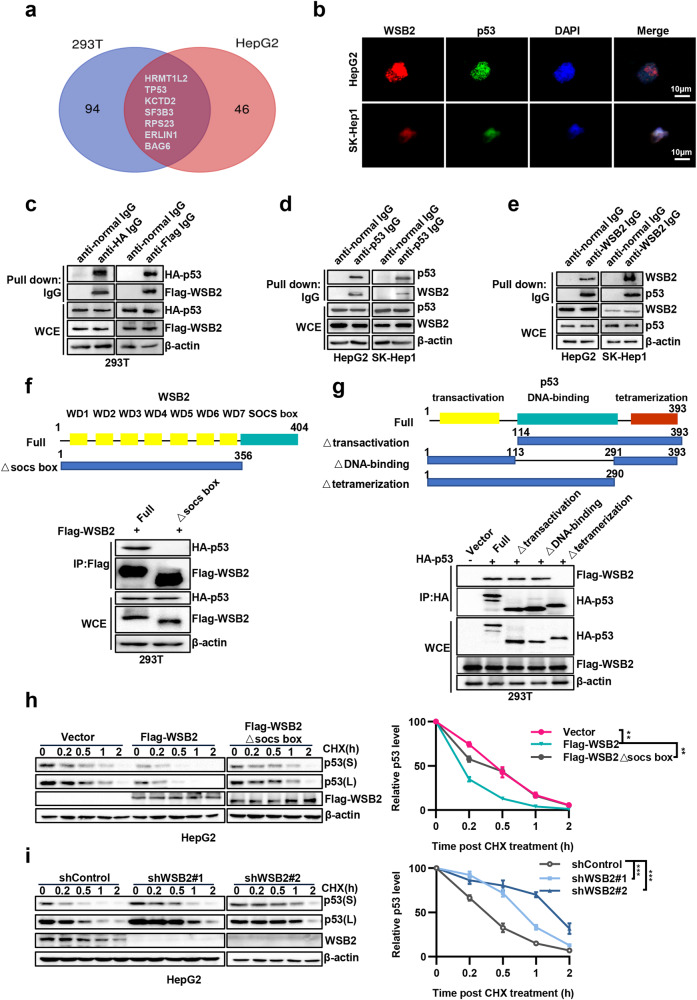
WSB2 directly interacts with p53 and destabilizes p53. **a** Venn diagram showing the potential substrates of WSB2 identified by co-IP (coimmunoprecipitation) and mass spectrometry in HEK293T cells (blue) and in HepG2 cells (red). **b** Immunofluorescence staining showing the colocalization of endogenous WSB2 and p53. Scale bar, 10 μm. **c** Exogenous WSB2 interacted with exogenous p53 in HEK293T cells. HEK293T cells were transfected with Flag-WSB2 plasmids or HA-p53 plasmids for 48 hours and then treated with MG132 for 5 hours before being collected. **d**, **e** The interaction between endogenous WSB2 and p53 in HepG2 and SK-Hep1 cells was analyzed by Co-IP. HepG2 and SK-Hep1 cells were treated with MG132 for 5 hours before harvest. **f**, **g** WSB2 bound to p53 tetramerization. 293 T cells were transfected with Flag-WSB2 or its mutant ΔSOCS plasmids for 48 hours or HA-p53 or its mutants with deletion of transactivation (Δtransactivation), DNA-binding (ΔDNA-binding), or tetramerization (Δtetramerization) for 48 hours and then lysed. Cell lysates were added to the indicated antibodies and Protein A/G PLUS-Agarose. **h**, **i** The half-life of p53 controlled by WSB2 was analyzed by Western blotting. HepG2 cells were transfected with Flag-WSB2 or its mutant ΔSOCS loss for 48 hours or shRNA targeting WSB2 (shWSB2) for 48 hours and then exposed to 100 μg/ml cycloheximide (CHX) for 3 hours. The intensity of p53 expression for each band was normalized to that of β-actin. S, Short exposure; L, long exposure. Data are shown as the means ± SEMs. Statistical significance was assessed by two-way analysis of variance. **p < 0.01, ***p < 0.001.

To examine whether WSB2 binds to p53, we first analyzed the potential substrates of WSB2 by immunoprecipitation and mass spectrometry. Ninety-four proteins were identified as candidates for interaction with WSB2 in a pull-down assay using anti-WSB2 antibody in HEK293T cells, and 46 proteins were identified in HepG2 cells (Supplementary Tables 7, 8). We made a chart showing the overlapping proteins to identify potential WSB2 substrates. There were 7 overlapping proteins, including p53, suggesting that p53 might be a potential substrate of WSB2 (Fig. 3a). To validate the interaction between WSB2 and p53, we analyzed the colocalization of WSB2 and p53 by immunofluorescence staining. Endogenous WSB2 and p53 were mainly colocalized in the nucleus (Fig. 3b). To further assess the interaction between WSB2 and p53, we performed a co-IP experiment and found that endogenous WSB2 was able to interact with endogenous p53 in p53 wild-type HCCs (Fig. 3d, e). Furthermore, exogenous WSB2, but not its mutant without the SOCS box (ΔSOCS), interacted with exogenous p53 (Fig. 3c, f). These findings suggest that WSB2 binds with p53.

To map the WSB2-interacting domain on p53, we constructed HA-tagged p53 deletion mutants, including p53 transactivation domain deletion (Δ1-113), p53-DNA binding domain deletion (Δ114-290), and p53 tetramerization domain deletion (Δ291-393). We found that p53 transactivation domain deletion (Δ1-113) and p53-DNA binding domain deletion (Δ114-290) retained the ability to interact with WSB2, whereas p53 with tetramerization domain deletion did not (Fig. 3g), suggesting that the amino acid sequence comprising residues 291 to 393 of p53 (tetramerization domain of p53) contains the WSB2-binding domain. We next examined whether WSB2 affects p53 stability. A time-course analysis following cycloheximide (CHX) blockade of protein synthesis indicated that overexpression of WSB2 but not its mutant (WSB2ΔSOCS box) shortened the half-life of p53 in HepG2 and SK-Hep1 cells, whereas silencing WSB2 extended the half-life (Fig. 3h, i; Supplementary Fig. 7a, b).

### WSB2 mediates K48-linked p53 ubiquitination at K291/292, and this effect is independent of Mdm2

**Fig. 4:**
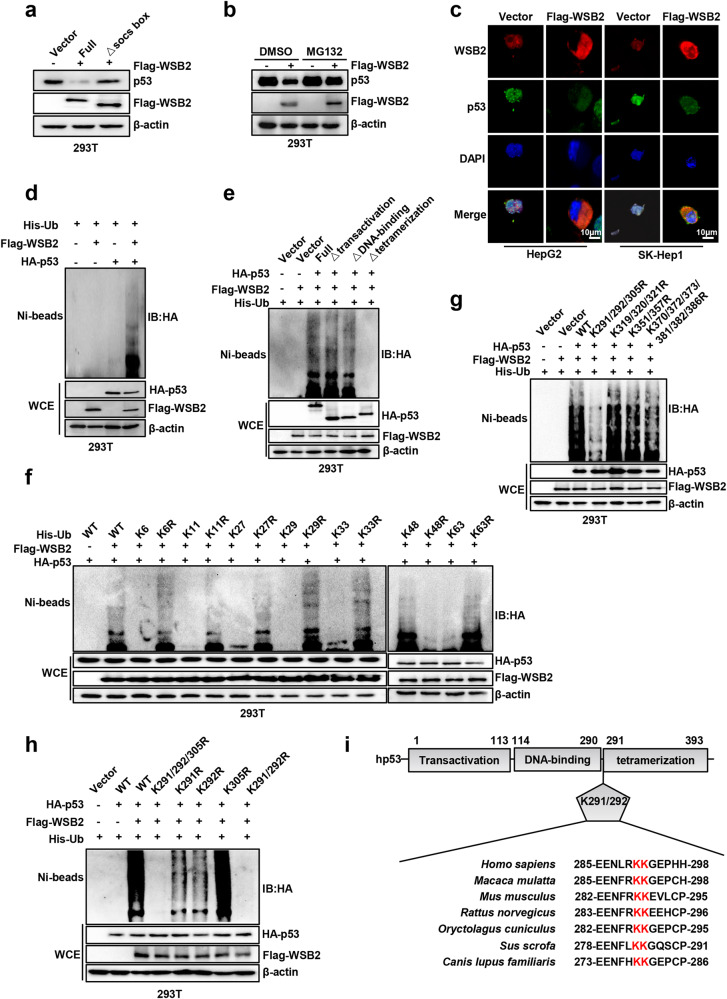
WSB2 promotes K48-linked ubiquitination of p53 at K291/292 sites. **a** WSB2 but not its mutant (ΔSOCS) downregulated p53 expression. HEK293T cells were transfected with Flag-WSB2 or its mutant ΔSOCS loss for 48 hours. **b** The proteasome inhibitor MG132 (10 μm) significantly blocked WSB2-mediated downregulation of p53 expression in HEK293T cells. **c** Immunofluorescence staining showing that upregulated WSB2 promotes the nuclear export of p53. Cells were transfected with or without Flag-WSB2 for 48 hours and then stained with anti-Flag or anti-p53 antibodies. Scale bar, 10 μm. **d** The in vivo ubiquitination assay indicated that WSB2 ubiquitinated wild-type p53. HEK293T cells were transfected with the indicated plasmids for 48 hours, followed by MG132 treatment for 5 hours before lysis. The lysates were then subjected to pull-down using Ni-NTA beads and Western blotting analysis with the indicated antibodies. **e** The full-length p53, p53 Δtransactivation and ΔDNA-binding mutants but not the Δtetramerization mutant were ubiquitinated by WSB2. HEK293T cells were transfected with ubiquitin (Ub) and WSB2 with wild-type p53 or its deleted mutants for 48 hours, followed by MG132 treatment for 5 hours before lysis. **f** WSB2 mediated K48-linked ubiquitination of p53. HEK293T cells were transfected with WSB2, p53 and wild-type ubiquitin (Ub) or its mutants for 48 hours, followed by MG132 treatment for 5 hours before lysis. **g** The mutation of p53 at the K291/292/305 sites completely blocked WSB2-mediated p53 polyubiquitination. **h** WSB2 catalyzed p53 polyubiquitination at K291/292 sites. **i** Amino acid sequences of p53 with K291/292 ubiquitination sites are highly conserved in the various species.

WSB2 shortened the half-life of p53. Consistently, we found that WSB2 but not its mutant (ΔSOCS) downregulated p53 expression (Fig. 4a). We speculated that WSB2 might degrade p53 via the ubiquitin‒proteasome system. As expected, the proteasome inhibitor MG132 significantly blocked WSB2-mediated downregulation of p53 expression (Fig. 4b). Immunofluorescence staining showed that upregulation of WSB2 promoted the nuclear export and degradation of p53 (Fig. 4c). Moreover, the in vivo ubiquitination assay indicated that WSB2 ubiquitinated wild-type p53 in both 293 T cells and HepG2 cells, whereas the WSB2 mutant (ΔSOCS) lost this ability (Fig. 4d, Supplementary Fig. 7c,d). In line with the interaction of WSB2 with p53 tetramerization shown in Fig. 3g, loss of the p53 tetramerization domain completely blocked WSB2-mediated p53 polyubiquitination (Fig. 4e). Furthermore, we found that Mdm2 did not attenuate WSB2-mediated p53 polyubiquitination (Supplementary Fig. 7e, f). Taken together, these results indicate that WSB2 promotes p53 polyubiquitination in HCC cells independent of Mdm2.

ElonginB/C-Cullin2/5-SOCS-box (ECS) members usually catalyze Lys63-linked polyubiquitination in the target protein to cause protein accumulation and activation^24^, whereas Lys48- or Lys11-linked polyubiquitination induces proteasomal degradation^25^. Lys27- or Lys29-linked polyubiquitination is involved in the immune response and signal transduction^26^, whereas Lys6- or Lys33-linked polyubiquitination are involved in autophagy^27^. To characterize the type of WSB2-mediated p53 ubiquitination, various mutants of His-ubiquitin vectors were constructed. Ubiquitin lysine residues (K6, K11, K27, K29, K33, K48, and K63) were substituted with arginine at all sites except the indicated site to generate mutants (K6R, K11R, K27R, K29R, K33R, K48R, and K63R). As expected, we found that WSB2 mediates K48-linked polyubiquitination of p53, since p53 ubiquitination occurred only in the K48 ubiquitin group (Fig. 4f).

Recent studies reported that ubiquitination of p53 at the K291/292/305, K319/320/321, K351/357, or K370/372/373/381/382/386 sites could cause p53 proteasomal degradation^28,29^. To explore the p53 ubiquitination site mediated by WSB2, a series of vectors with lysine-to-arginine substitutions in the p53 tetramerization region (K291/292/305R, K319/320/321R, K351/357R, and K370/372/373/381/382/386R) were constructed. Cells were transfected with WSB2 plasmids with the indicated p53 mutations. We observed that mutation of the K291/292/305 site to alanine (K291/292/305R) in p53 nearly completely blocked WSB2-mediated p53 polyubiquitination, whereas the K319/320/321R, K351/357R, and K370/372/373/381/382/386R mutants in p53, similar to wild-type p53, significantly promoted p53 polyubiquitination (Fig. 4g). Furthermore, the K291R or K292R mutant partly blocked WSB2-mediated p53 polyubiquitination, whereas the K291/292R mutant completely abolished this action, indicating that WSB2 catalyzed p53 polyubiquitination at the K291/292 site (Fig. 4h). The p53 K291/292 sites are evolutionarily conserved throughout various species (Fig. 4i). In summary, these results indicate that WSB2 promotes the K48-linked polyubiquitination of p53 at the Lys291 and Lys292 sites, and this process is independent of Mdm2.

### WSB2 activates the p53-IGFBP3-AKT-mTOR signaling pathway to promote the development of HCC

**Fig. 5:**
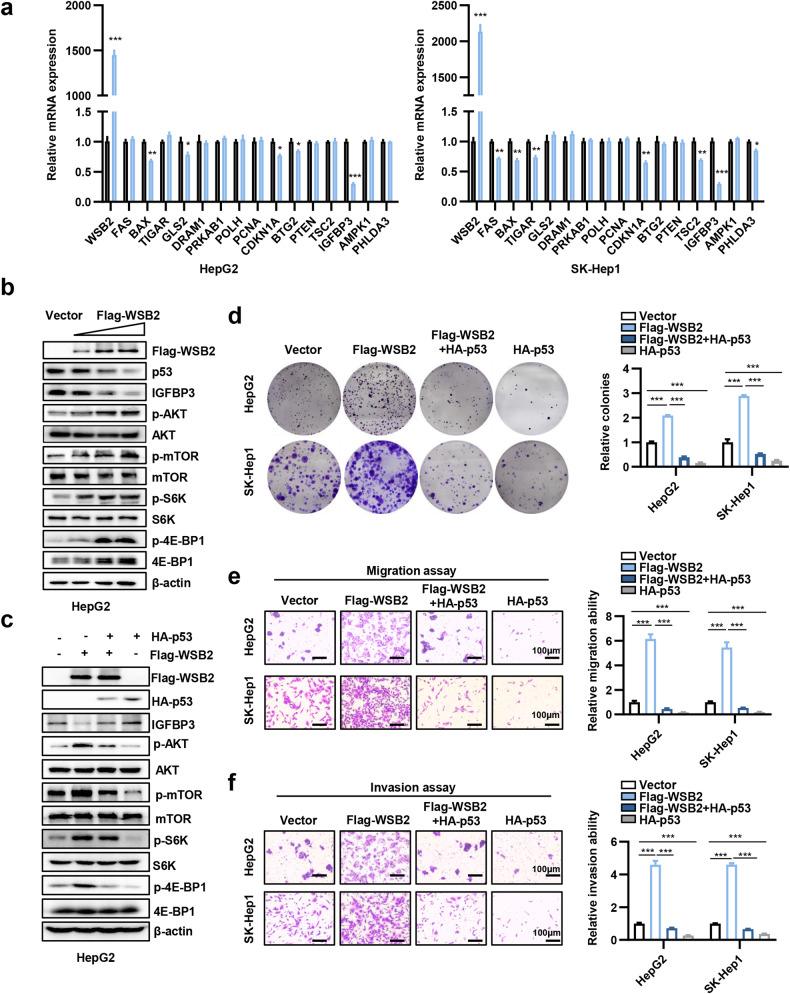
WSB2 activates the p53-IGFBP3-AKT-mTOR signaling pathway and promotes cell proliferation and metastasis in HCC. **a** The p53 downstream genes were assessed by qPCR after WSB2 overexpression in HepG2 and SK-Hep1 cells. **b**, **c** HepG2 cells were transfected with the indicated plasmids for 48 hours and then analyzed by Western blotting with the indicated antibodies. **d** Representative images of cell colonies and quantification of colony numbers in WSB2-overexpressing cells with or without the HA-p53 vector. *n* = 3. **e**, **f** Representative images and quantification of cell migration (**e**) and invasion (**f**) in WSB2-overexpressing cells with or without p53 overexpression. *n* = 3. Scale bar, 100 μm. Data are shown as the means ± SEMs. Statistical significance was assessed by Student’s two-tailed t test (**a**) or one-way ANOVA with Tukey’s multiple comparison test (**d**–**f**). *p < 0.05, **p < 0.01, ***p < 0.001.

Given that WSB2 promoted p53 degradation, we wanted to know the potential downstream target of p53 involved in WSB2-mediated cell proliferation and metastasis. The expression of p53 downstream genes FAS, BAX, TIGAR, GLS2, DRAM1, PRKAB1, POLH, PCNA, CDKN1A, BTG2, PTEN, TSC2, IGFBP3, AMPK1 and PHLDA3 was assessed by qPCR after WSB2 overexpression in HepG2 and SK-Hep1 cells. The results indicated that the mRNA expression of BAX, CDKN1A, and IGFBP3 was significantly downregulated by WSB2 in both HepG2 and SK-Hep1 cells (Fig. 5a). Among them, IGFBP3 was strongly downregulated upon overexpression of WSB2 compared with BAX and CDKN1A, suggesting that IGFBP3 may play a key role in WSB2-mediated cell proliferation and metastasis. Therefore, we studied the role of IGFBP3 in WSB2/p53-driven HCC proliferation and metastasis. As IGFBP3 is a key protein involved in the AKT/mTOR pathway in HCC^30,31^, we analyzed the effect of WSB2 on the IGFBP3/AKT/mTOR pathway. We found that WSB2 reduced p53 and IGFBP3 expression and then activated AKT, mTOR and mTOR downstream targets S6K (Thr389) and 4E-BP1 (Thr37/46) in TP53 wild-type cells (HepG2 and SK-Hep1) but not in TP53 mutant-type cells (PLC/PRF/5) (Fig. 5b, Supplementary Fig. 8a, c). To further validate WSB2-mediated AKT/mTOR activation via degradation of p53, we transfected WSB2 plasmids with or without p53 plasmids in HepG2 and SK-Hep1 cells. WSB2 significantly downregulated IGFBP3 and upregulated the active forms of AKT and mTOR (downstream effectors p-S6K and p-4EBP1), whereas overexpression of p53 dramatically blocked these actions (Fig. 5c, Supplementary Fig. 8b). To further explore the role of p53 in WSB2-triggered HCC, HepG2 and SK-Hep1 cells were transfected with WSB2 plasmids with or without p53 plasmids. WSB2-mediated cell proliferation, migration and invasion were significantly attenuated by p53 (Fig. 5d–f). In addition, the functions of IGFBP3 in WSB2-triggered HCC were further validated. Overexpression of IGFBP3 dramatically blocked WSB2-mediated AKT/mTOR activation and attenuated WSB2-mediated cell proliferation, migration and invasion (Supplementary Fig. 8d–g). As CDKN1A, a cell cycle-related gene, was downregulated upon overexpression of WSB2, we analyzed the effect of WSB2 knockdown on the cell cycle. We found that silencing WSB2 significantly induced cell cycle arrest at G1 phase in p53 wild-type HCC cells (Supplementary Fig. 9a). Furthermore, WSB2 decreased the expression of the apoptosis biomarker BAX. Consistent with this finding, silencing WSB2 induced apoptosis in p53 wild-type HCC cells (Supplementary Fig. 9b). These findings indicated that high expression of WSB2 in TP53 wild-type HCC cells promoted cell proliferation and inhibited apoptosis. Taken together, these results indicate that WSB2 promotes HCC via the p53/IGFBP3/AKT/mTOR axis.

### Everolimus reverses WSB2-driven hepatocellular carcinogenesis in vitro and in vivo

**Fig. 6:**
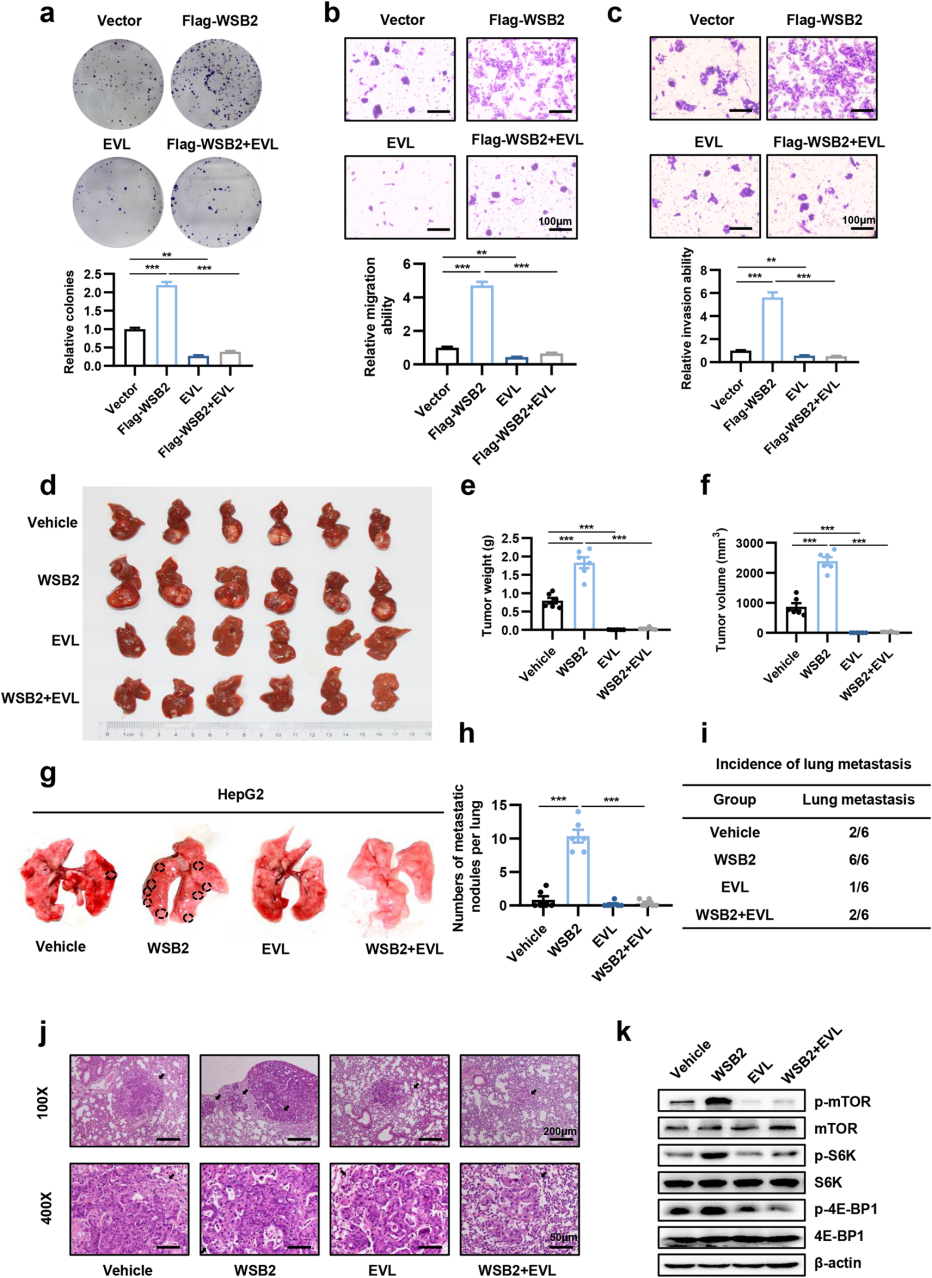
Everolimus blocks WSB2-driven hepatocellular carcinogenesis in vitro and in vivo. **a**–**c** Effect of everolimus (EVL) on the proliferation (**a**), migration (**b**) and invasion (**c**) of WSB2-overexpressing HepG2 cells in vitro. After transfection with the Flag-WSB2 vector for 24 hours, the cells were treated with 100 nM everolimus (EVL) for 48 hours and collected. Scale bar, 100 μm. **d**–**f** Effect of everolimus (EVL) on WSB2 overexpression-driven HCC tumorigenesis in vivo. Orthotopic xenograft tumor models were established and intravenously injected with everolimus (5 mg/kg, i.p., twice per week) for 5 weeks. Tumor weight and tumor volume were calculated. *n* = 6 mice/group. **g**–**j** Effect of everolimus (EVL) on the invasion of WSB2-overexpressing HepG2 cells in vivo. An experimental mouse model of lung metastasis was established by tail-vein injection of WSB2-overexpressing HepG2 cells. Five days later, the mice were intravenously injected with everolimus (5 mg/kg, i.p., twice per week) for 7 weeks. Tumors were imaged (**g**); metastatic lung nodules were counted (**h**); and the incidence of lung metastases was calculated (**i**). *n* = 6 mice/group. Representative H&E staining images showing lung metastatic nodules (indicated by black arrow, **j**). **k** Representative protein levels of key mTOR markers in xenograft tumor models treated with or without everolimus (EVL, 5 mg/kg) were detected by Western blotting. Data are shown as the means ± SEMs. Statistical significance was assessed by one-way ANOVA with Tukey’s multiple comparison test (**a**–**c**, **e**, **f**, **h**) or χ2 test (**i**). **p < 0.01, ***p < 0.001.

Everolimus (EVL), a specific oral mTOR inhibitor, has been approved by the U.S. Food and Drug Administration to treat various cancers, such as breast cancer, pancreatic cancer and renal cell carcinoma^32^. Given that everolimus showed antitumor activity in preclinical models of pancreatic cancer and breast cancer metastasis^33^, we inferred that everolimus may be a therapeutic agent for WSB2/mTOR axis-mediated HCC progression. We then investigated the therapeutic effect of the mTOR inhibitor everolimus on hepatocarcinogenesis and metastasis triggered by WSB2 in vitro and in vivo. We found that everolimus could significantly inhibit WSB2-mediated colony formation, cell migration and invasion in HepG2 cells (Fig. 6a–c). Subsequently, WSB2-overexpressing HepG2 cells were cultured, transplanted into the hypodermis of nude mice, and then retransplanted into the livers of 24 male nude mice to establish an orthotopic HCC xenograft mouse model. Compared to the vehicle, the mTOR inhibitor everolimus (5 mg/kg, i.p., twice per week) significantly reduced the tumor weight and tumor volume (Fig. 6d–f). Furthermore, we established a xenograft mouse model of lung metastasis via tail-vein injection of WSB2-overexpressing HepG2 cells. We observed that everolimus significantly blocked lung metastasis of HCC, as indicated by a decrease in the incidence of lung metastasis and the number of metastatic nodules in the everolimus treatment group compared with the control group (Fig. 6g–j). To confirm the effect of everolimus on the inhibition of WSB2-triggered HCC in a xenograft mouse model, Everolimus-treated tumors were isolated, and mTOR signals were analyzed by Western blotting. mTOR activity was significantly reduced in Everolimus-treated tumors compared with WSB2-overexpressing tumors, as indicated by the decrease in p-mTOR and its downstream targets p-S6K and p-4EBP1 (Fig. 6k). Taken together, these results indicate that targeting mTOR effectively inhibits WSB2-triggered HCC tumorigenesis and metastasis.

### Elevated WSB2 expression is associated with wild-type p53 status and active mTOR in HCC patients

**Fig. 7:**
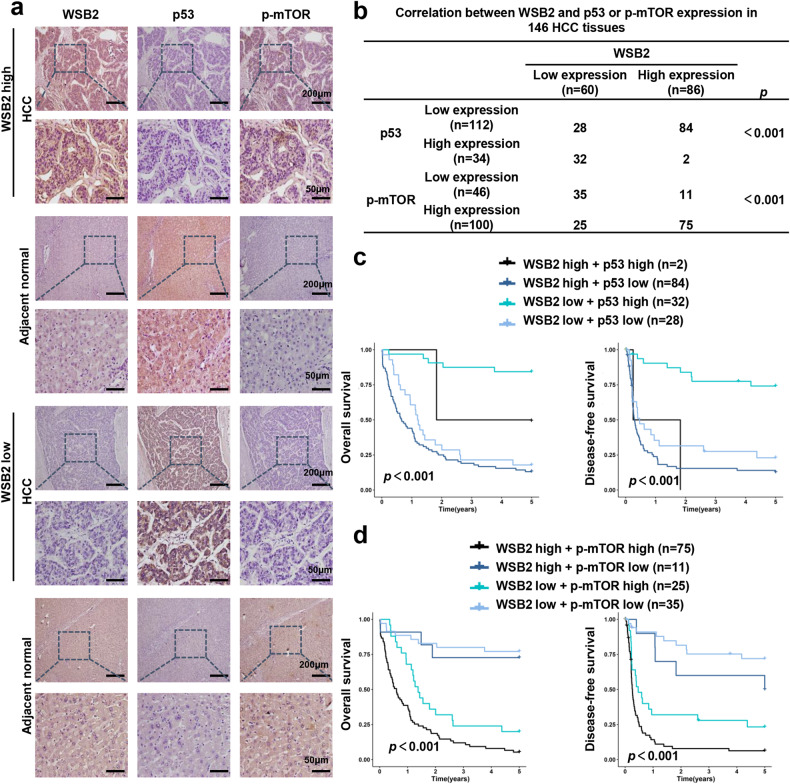
High WSB2 expression is associated with decreased p53 expression and increased mTOR activity in human HCC tissues. **a** Representative immunohistochemical images showing the expression of WSB2, p53, and p-mTOR (Ser2448) in HCC samples and adjacent normal liver tissues. Scale bar, 200 μm or 50 μm. **b** Correlation between WSB2 and p53 or p-mTOR in HCC tissues. *n* = 146. **c** The association of WSB2 and p53 expression with overall survival time and disease-free survival time in HCC patients. **d** The association of WSB2 and p-mTOR expression with overall survival time and disease-free survival time in HCC patients. Statistical significance was assessed by the χ2 test (**b**) or log-rank test (**c**, **d**).

To explore the correlation between WSB2, wild-type p53 and p-mTOR levels in human HCC tissues, the expression of WSB2, wild-type p53 and p-mTOR in 146 paired HCC samples was examined by immunohistochemistry (Fig. 7a). In total, 57. 5% (84/146) of HCC tissues had significantly higher WSB2 expression and lower p53 expression than normal tissues according to the staining rate, suggesting possible p53 degradation by WSB2 in HCC (Fig. 7b). Furthermore, 51.4% (75/146) of HCC tissues had higher WSB2 and p-mTOR expression than normal tissues, indicating that WSB2 activates mTOR (Fig. 7b). Patients with high WSB2 and low p53/high p-mTOR expression profiles had a worse prognosis than those with low WSB2 and high p53/low p-mTOR expression profiles (Fig. 7c, d). These data indicated that WSB2 expression is negatively associated with wild-type p53 expression and positively associated with mTOR activation in HCC patients.

## Discussion

Hepatocarcinogenesis is a complex multistep, highly precisely regulated biological process including genetic and epigenetic alterations^34^. These alterations lead to changes in tumor-related signaling pathways, accompanied by the activation of oncoproteins and dysregulation of tumor suppressors. Accumulating evidence shows that many structural or functional changes in E3 ligases are related to the progression of HCC^35^. Dysregulation of E3s contributes to improper assembly of protein complexes, accumulation of misfolded or dysfunctional proteins, and abnormal activation or inactivation of signaling factors, thus promoting the development and progression of many cancers, including HCC^36^. Herein, we report a new E3 ligase, WSB2, that is highly expressed in HCC and is associated with aggressive tumor phenotypes and a poor prognosis. WSB2 is an independent risk factor that can effectively predict the prognosis of patients after resection of HCC. WSB2 promotes HCC cell proliferation, migration and invasion in vitro and promotes tumor growth and lung metastasis in vivo. These results indicated that the E3 ligase WSB2 plays an important role in HCC tumorigenesis and development.

p53 is one of the most famous tumor suppressors^37^. In normal cells, the stability of the p53 protein is extremely poor, with a half-life of only 20 minutes. Most p53 protein can be degraded in the cytoplasm within a short time after synthesis^38^. Ubiquitination is the most important posttranslational modification pathway responsible for p53 protein degradation^8^. Ubiquitination can inhibit the functional activity of p53, promote the nuclear export of p53 and mediate its degradation through the proteasome pathway in the cytoplasm^9^. A variety of E3 ligases can specifically recognize and mediate the ubiquitination of p53^35^. The most well-known is Mdm2^14^. Mdm2 can not only regulate the ubiquitination and degradation of p53 but also block the transcriptional activity of p53 by binding to the transcriptional activation domain of p53 in the form of a homolog or heterodimer^39^. Mdm2 modifies p53 at six lysine residues (K370, K372, K373, K381, K382, and K386)^29^. Our work demonstrated that WSB2 could directly interact with p53 and destabilize p53 by promoting K48-linked ubiquitination of p53 at the Lys291 and Lys292 sites. These residues differ from the lysine residue, which is mediated by Mdm2. Moreover, we found that WSB2-mediated p53 ubiquitination is independent of Mdm2. Given that the ability of some small molecular inhibitors targeting Mdm2, such as Nutlin and MI-319, to reactivate p53 functions is limited^40,41^, herein, we provided evidence that WSB2 could be a potential drug target for restoring p53 functions.

Insulin-like growth factor binding protein-3 (IGFBP3) is a major carrier for insulin-like growth factors (IGFs) in circulation and is upregulated by p53 at the transcriptional level^31,42^. Previous studies revealed that two p53 binding sites, Box A and Box B, were identified within the IGFBP3 gene^31^, and p53 mutants lost the ability to activate IGFBP3^42^. IGFBP3, which is induced by p53, has been found to negatively regulate the IGF-1/AKT pathway by binding to free IGF-1^31^. The loss of AKT activity increases TSC1-TSC2 activity, which in turn decreases mTORC1 activity^30^. Thus, p53 activates IGFBP3, in turn shutting down evolutionarily conserved IGF-1/AKT and mTOR pathways, which play critical roles in the regulation of cell proliferation, survival, and energy metabolism^43^. Consistent with previous studies, our results demonstrated that upregulation of WSB2 decreased p53 expression and then inhibited IGFBP3 expression, followed by activation of the AKT/mTOR pathway.

Abnormal activation of the mammalian target of rapamycin (mTOR) pathway is observed in 40–50% of patients with HCC. mTOR activation in HCC strongly correlates with poor tumor differentiation, poor prognosis and early tumor recurrence^44^. Everolimus is an mTOR inhibitor that is designed for oral administration. Several large-scale randomized controlled trials (RCTs) have demonstrated the survival benefits of everolimus for solid cancers^33,45,46^. In phase I/II clinical trials, everolimus showed antitumor activity and was well tolerated in patients with advanced HCC^47^. A systematic review and meta-analysis showed that everolimus (EVL) benefitted 1-, 2-, 3-, and 5-year OS in patients undergoing liver transplantation for HCC^48^. However, the response rate of single everolimus to HCC is low^47^. Therefore, combination therapy with everolimus or searching for subtypes suitable for everolimus treatment may be necessary^49^. A previous study confirmed that p53 restoration markedly improves the sensitivity of HCC cells to everolimus^50^. Various methods have been developed to reactivate p53 functions. Some small molecular inhibitors targeting specific E3 ligases, such as Nutlin and MI-319, have shown high binding potency and selectivity for Mdm2 in the treatment of HCC and other cancers^40,41^. Our research revealed that everolimus reversed the promoting effects of WSB2 overexpression on hepatocellular carcinogenesis in vitro and in vivo, which indicated that HCC patients with high WSB2 expression may benefit from everolimus treatment. However, the questions of how effective everolimus monotherapy is and whether it is necessary to combine everolimus with molecular inhibitors targeting WSB2 still deserve further study.

Several important limitations of our study must be acknowledged. Although we have demonstrated that WSB2 is highly expressed in HCC tissues and that high WSB2 expression is correlated with adverse clinical features, this phenomenon was mainly observed in the wild-type TP53 group. The effect of WSB2 on TP53 wild-type and mutant types and its clinical implications still need further validation in patients. First, we found that the large majority of young patients were in the WSB2-high group, and this phenomenon was also observed for the TP53-mutant HCC group. This may be related to the limited number of patients over the age of 60 years old in our study. This may also be due to the influence of individual age on WSB2 expression in HCC. The increased incidence of excessive alcohol consumption or obesity in young patients may also be responsible for the increased expression of WSB2. Second, the analyses performed on the relationships between WSB2 and clinicopathological parameters are based on cross-sectional data rather than a prospective study. The poor prognostic effect of WSB2 needs to be further validated in patients. Third, due to the low p53 mutation rate, there was a limited number of TP53-mutant patients. In TP53-mutated HCC patients, the survival curves of the high and low WSB2 groups intersected, and the survival curve for the WSB2-low group showed a sharp decline at an early time point. These findings may be attributed to the limited number of total HCC patients, which may lead to the possibility of biased interpretation.

**Fig. 8:**
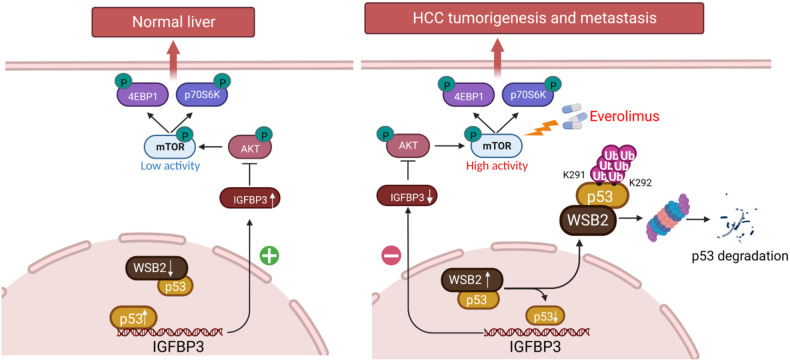
The proposed model depicts the role of WSB2 in HCC carcinogenesis. Under normal conditions, a low level of WSB2 maintains p53 at a low level of ubiquitination and degradation, which causes p53 at a high level and IGFBP3-dependent inhibition of AKT/mTOR hyperactivation, leading to normal cell growth (left panel). In HCC, high expression of WSB2 promotes p53 degradation and reduces IGFBP3 to block ATK/mTOR hyperactivation, leading to HCC tumorigenesis. Targeting mTOR with everolimus, a specific oral inhibitor of mTOR, significantly blocks HCC tumorigenesis and invasion (right panel).

In conclusion, our research demonstrated that WSB2 is frequently upregulated in HCC. Elevated WSB2 ubiquitinates p53 at the Lys291 and Lys292 sites via the K48-linked polyubiquitination pathway. WSB2 degrades p53, which leads to decreased IGFBP3 expression and increased AKT Ser 473 phosphorylation, subsequently activating mTOR signaling and contributing to HCC carcinogenesis and metastasis (outlined in Fig. 8). The WSB2/p53/IGFBP3/AKT/mTOR axis could be a potential therapeutic target for patients with HCC.

### Supplementary information

Supplementary data
