# Supplementary material for: Elevated expression of WSB2 degrades p53 and activates the IGFBP3-AKT-mTOR-dependent pathway to drive hepatocellular carcinoma
Source: Exp Mol Med. 2024 Jan 4;56(1):177–91. doi: 10.1038/s12276-023-01142-6 (PMC10834962; doi:10.1038/s12276-023-01142-6)
Supplement: Supplementary file 1 — Supplementary data [file 12276_2023_1142_MOESM1_ESM.pdf]

Supplementary information

Elevated expression of WSB2 degrades p53 and activates the IGFBP3-AKT-mTOR-dependent pathway to drive hepatocellular carcinoma

Xun Li[1,2#], Cheng-Cheng Zhang [1#], Xiao-Tong Lin[1], Jie Zhang[1], Yu-Jun Zhang[1], Hong-Qiang Yu[1], Ze-Yu Liu [1], Yi Gong[1], Lei-Da Zhang[1*], Chuan-Ming Xie[1,3*]

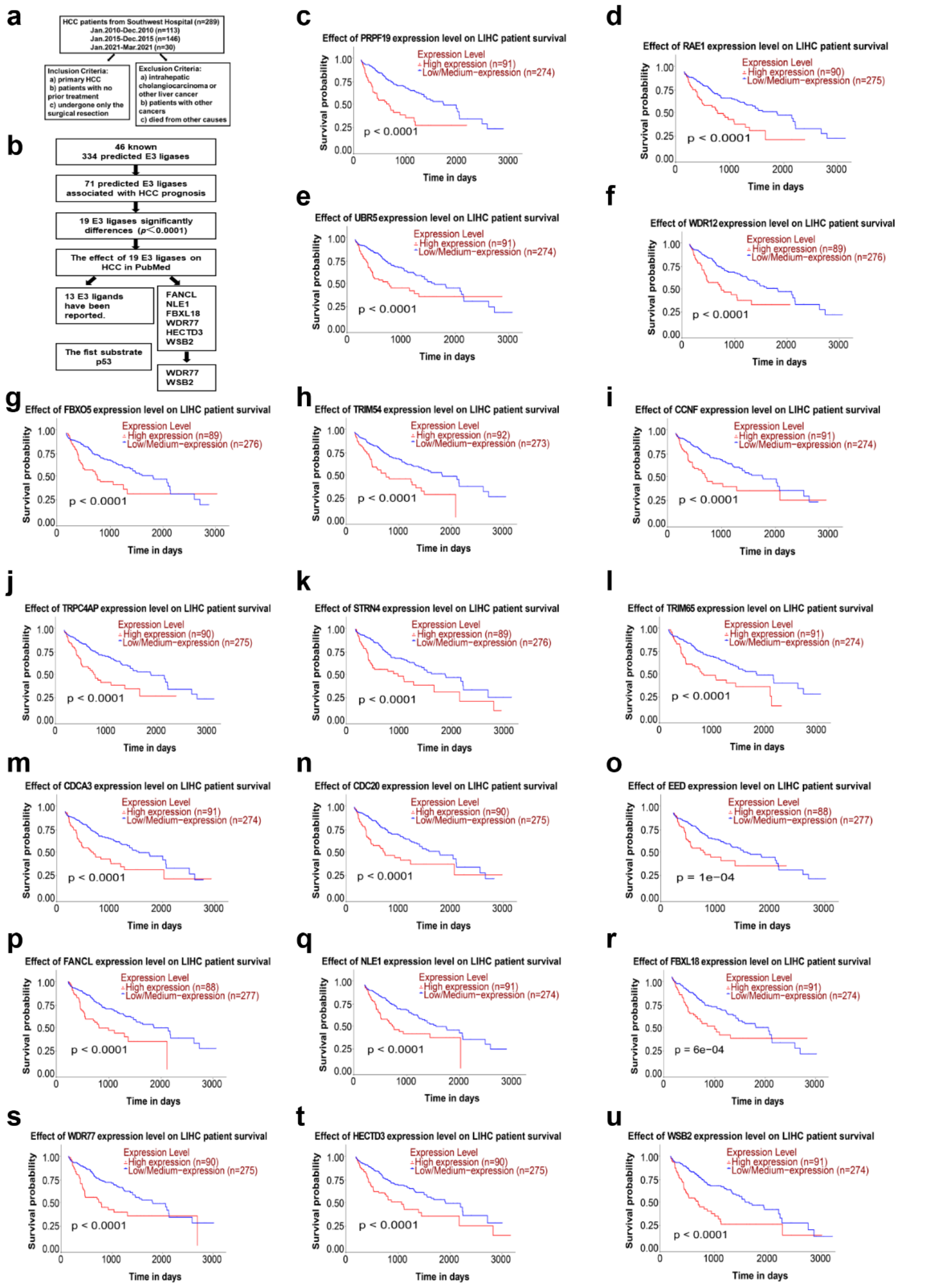

Supplementary Fig.1 The association between the predicted p53-related E3 ligases and overall survival in HCC patients.

(a) The inclusion and exclusion criteria of HCC patients **(b)** E3 ubiquitin ligases for p53 degradation were screened and predicated based on UbiBrowser database. **(c-o)**

The effect of 13 reported E3 ligases on survival in HCC patients. (c) PRPF19 (d) RAE1 (e) UBR5 (f) WDR12 (g) FBXO5 (h) TRIM54 (i) CCNF (j)TRPC4AP (k) STRN4 (l) TRIM65 (m) CDCA3 (n) CDC20 (o) EDD **(p-u)** The effect of six unreported E3 ligases on survival in HCC patients. (p) FANCL (q) NLE1 (r) FBXL18 (s) WDR77 (t) HECTD3 (u) WSB2.

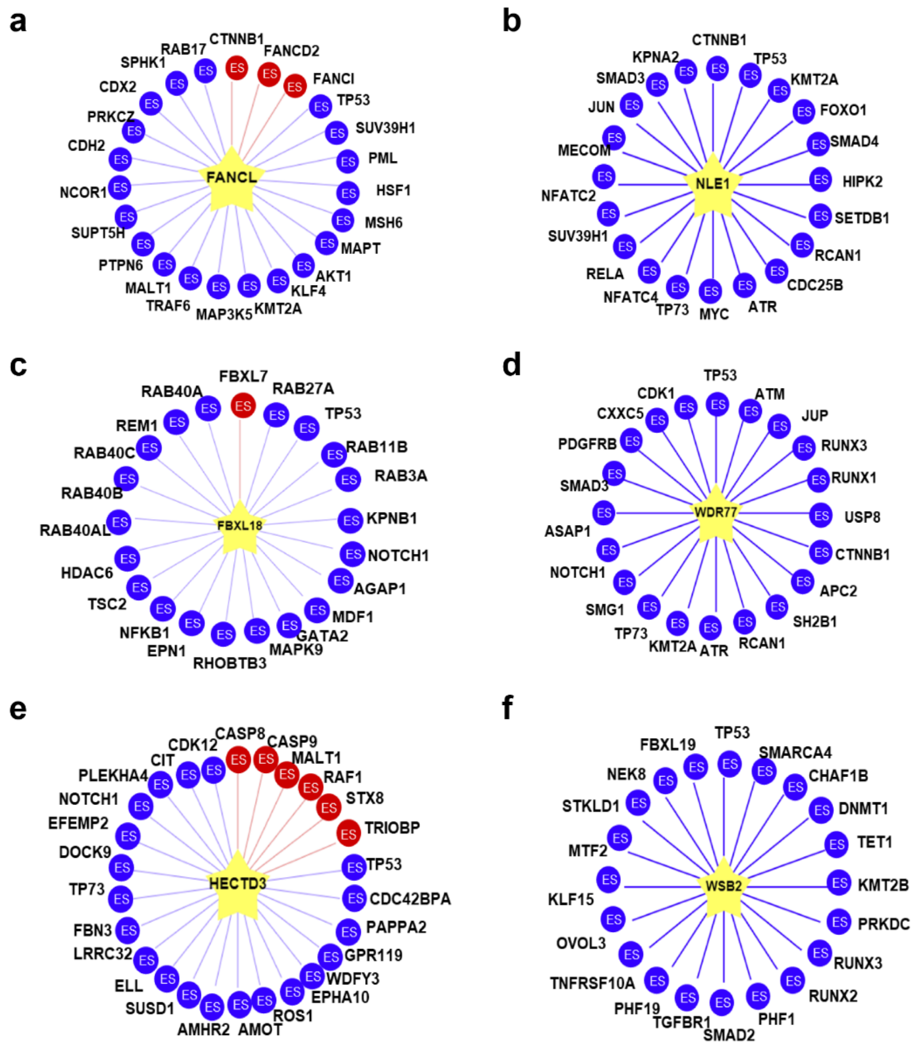

Supplementary Fig.2 The potential substrates of the six E3 ubiquitin ligases were analyzed based on UbiBrowser database.

(a) FANCL (b) NLE1 (c) FBXL18 (d) WDR77 (e) HECTD3 (f) WSB2

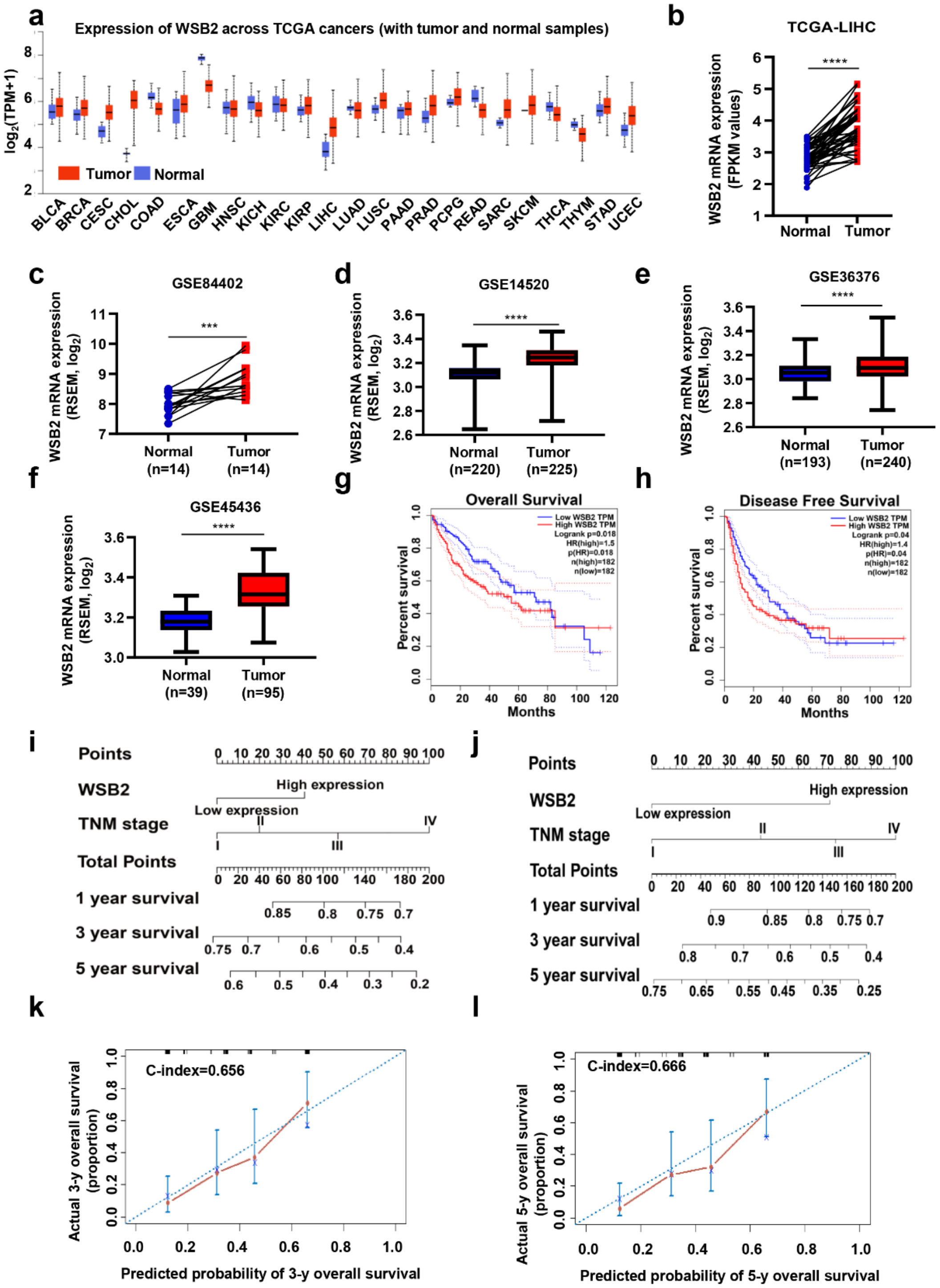

Supplementary Fig.3 High expression of WSB2 associates with poor prognosis in HCC patients.

(a) The mRNA levels of WSB2 in 24 cancer types were analyzed using UALCAN cancer database. **(b)** WSB2 expressions in 50 paired HCC and normal liver tissues from TCGA RNA-seq datasets. **(c-f)** WSB2 was upregulated in HCC samples compared with

normal liver tissues in GSE84402, GSE14520, GSE36376, and GSE45436 dataset. **(g,h)** Kaplan-Meier analysis assessed the overall survival and disease-free survival in patients with hepatocellular carcinoma in the TCGA cohort. **(i-l)** Nomograms for predicting the survival in patients with HCC after surgical resection and its predictive performance. The nomogram was used to predict overall survival based on the TCGA data (i) and IHC cohort (j); The calibration plot for the prediction of 3-y (k), 5-y (l) of overall survival in the IHC cohort was shown.

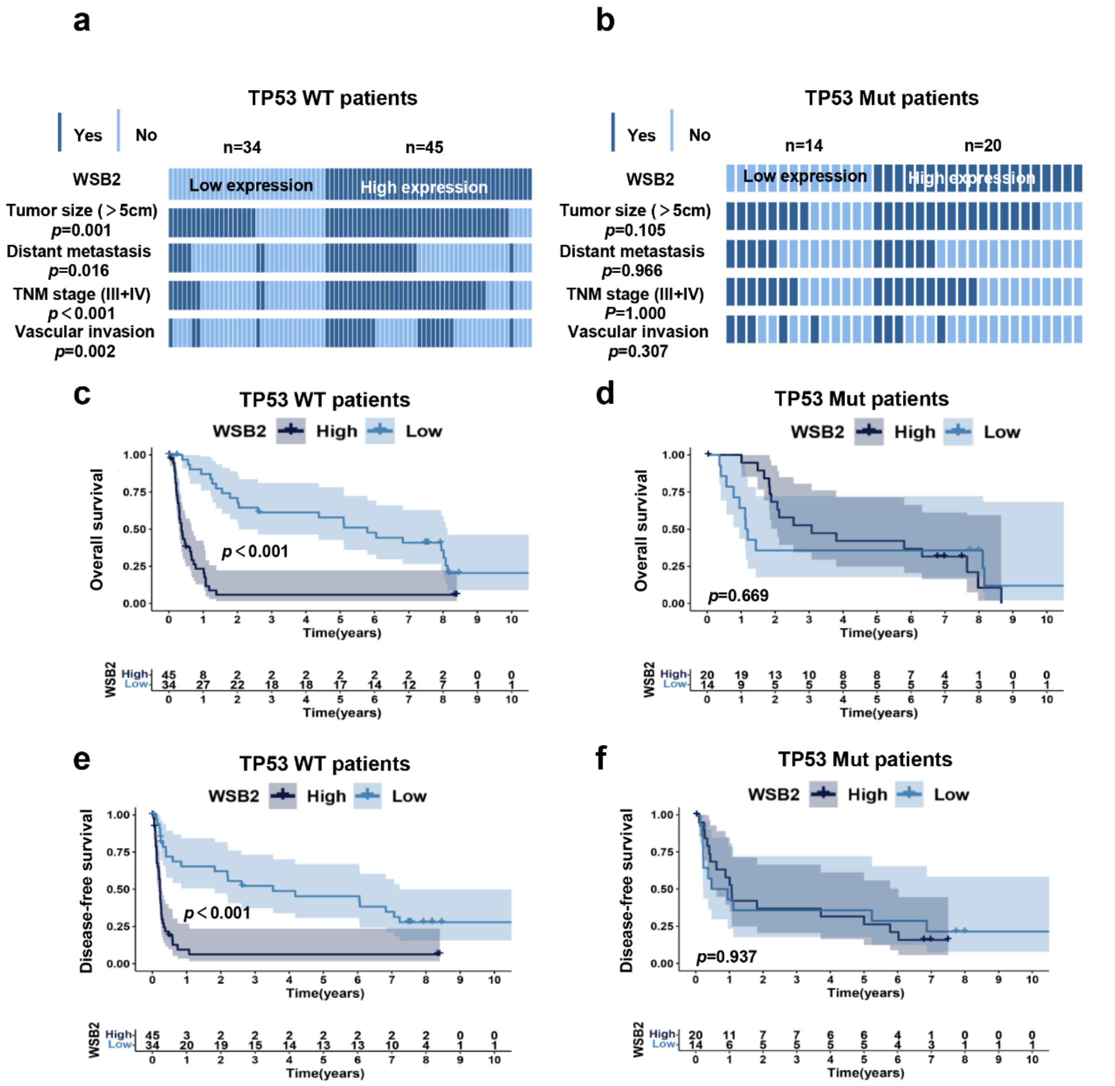

Supplementary Fig.4 The prognosis of WSB2 in HCC depends on TP53 gene status

(a,b) Chi-square test was used to investigate the association between WSB2 expression and tumor size, distant metastasis, vascular invasion or TNM stage in TP53 WT (a) and TP53 mutated (b) HCC patients. **(c-f)** The association between WSB2 expression and OS (overall survival) or DFS (disease free survival) in TP53 WT (c,e) and TP53 mutated (d,f) HCC patients were evaluated by Kaplan-Meier analysis, respectively. WT, wild-type.

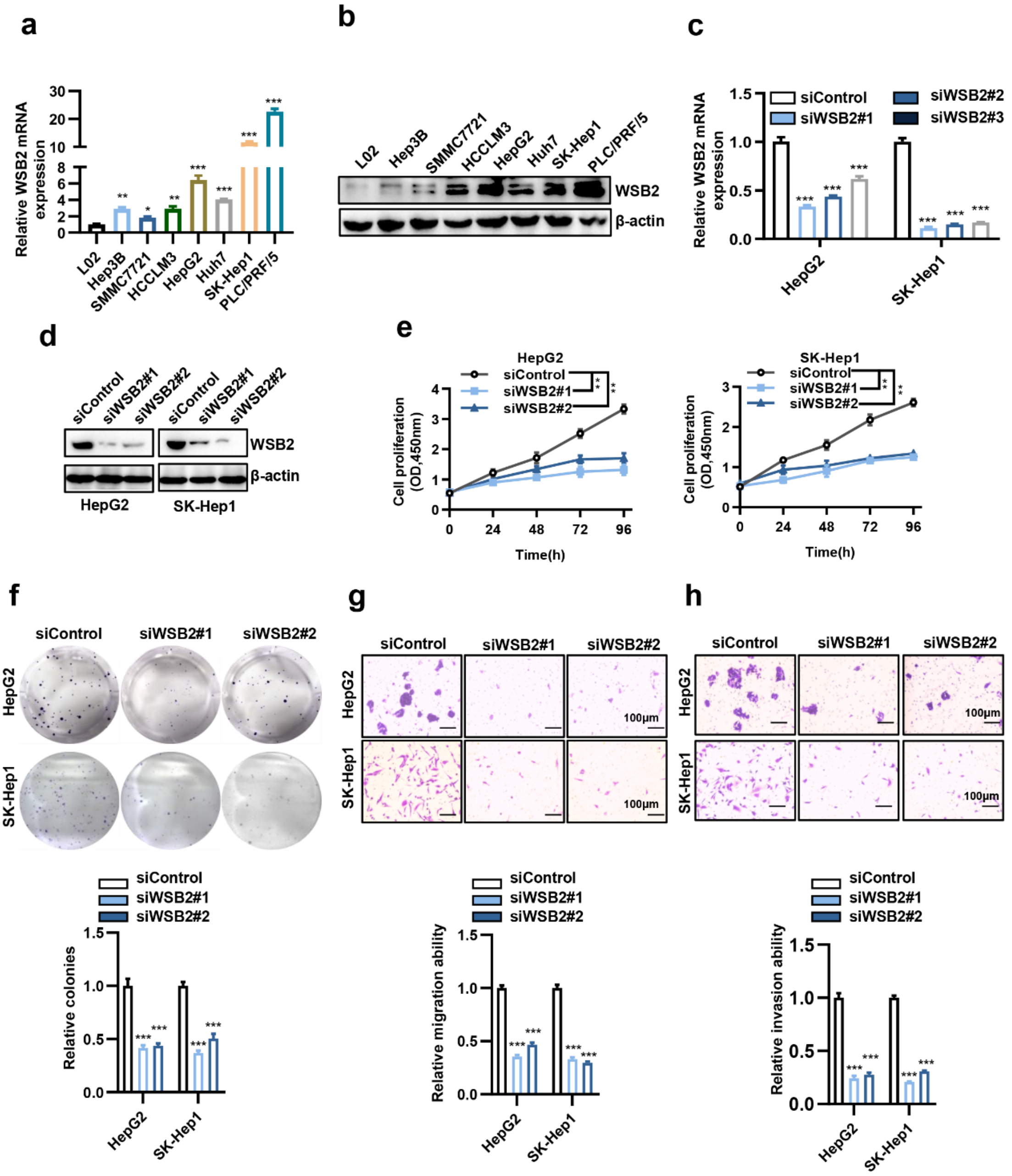

Supplementary Fig.5 WSB2 promotes HCC cell proliferation, migration and invasion *in vitro*.

(a) Relative WSB2 mRNA levels in 7 HCC cell lines, and normal human hepatic cell L02 were analyzed by qPCR. **(b)** Relative WSB2 protein levels in 7 HCC cell lines, and normal human hepatic cell L02 were analyzed by Western blotting. β-actin was used as loading control. **(c,d)** The efficiency of WSB2-knockdown in the indicated HCCs was detected by qRT-PCR (c) and Western blotting (d). **(e)** WSB2 knockdown inhibited cell proliferation in HCCs. Cells were transfected with siRNAs targeting

WSB2 (siWSB2) for different time intervals and analysed by CCK8 assay. **(f)** Representative images of cell colonies and quantification of colony numbers in WSB2-knockdown cells were shown**.** n = 3. **(g)** Cell migration was evaluated in WSB2-knockdown cells. n = 3. Scale bar, 100 μm. **(h)** Cell invasion was evaluated in WSB2-knockdown cells. n = 3. Scale bar, 100 μm. Data are shown as means ± SEMs. Statistical significance was calculated by one-way ANOVA with Tukey's multiple comparisons (a, c, f-h), two-way ANOVA with Bonferroni's multiple comparisons test (e). $*p < 0.05$, $**p < 0.001$, $***p < 0.001$.

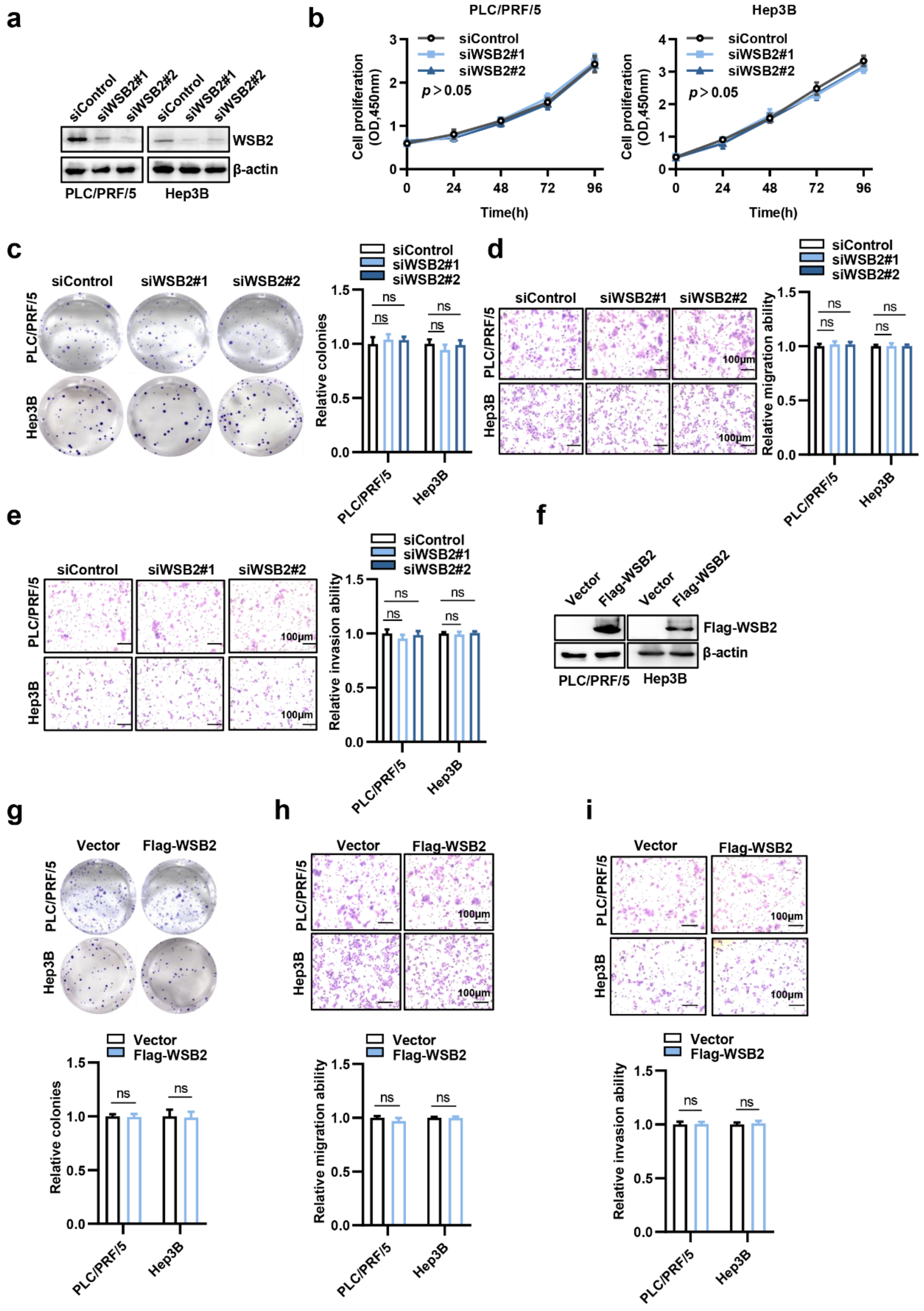

Supplementary Fig.6 WSB2 does not affect cell proliferation and metastasis in TP53 mutant-type cells

(a) The efficiency of WSB2-knockdown in TP53 mutant-type cells (PLC/PRF/5 and Hep3B) was detected by Western blotting. **(b)** TP53 mutant-type cells were transfected

with siRNAs targeting WSB2 (siWSB2) for different time intervals and analyzed by CCK8 assay. **(c)** Representative images of cell colonies and quantification of colony numbers in WSB2-knockdown cells were shown. n = 3. **(d)** Cell migration was evaluated in WSB2-knockdown cells. n = 3. Scale bar, 100 μm. **(e)** Cell invasion was analyzed in WSB2-knockdown cells. n = 3. Scale bar, 100 μm. **(f)** The efficiency of WSB2-overexpression in TP53 mutant-type cells was detected by Western blotting. **(g)** Representative images of cell colonies and quantification of colony numbers in WSB2-overexpression cells. n = 3. **(h)** Cell migration was evaluated in WSB2-overexpression cells. n = 3. Scale bar, 100 μm. **(i)** Cell invasion was evaluated in WSB2-overexpression cells. n = 3. Scale bar, 100 μm. Data are shown as means ± SEMs. Statistical significance was calculated by two-way ANOVA with Bonferroni's multiple comparisons test (b), one-way ANOVA with Tukey's multiple comparisons (c-e), or Student's t-test (g-i). ns, no significance.

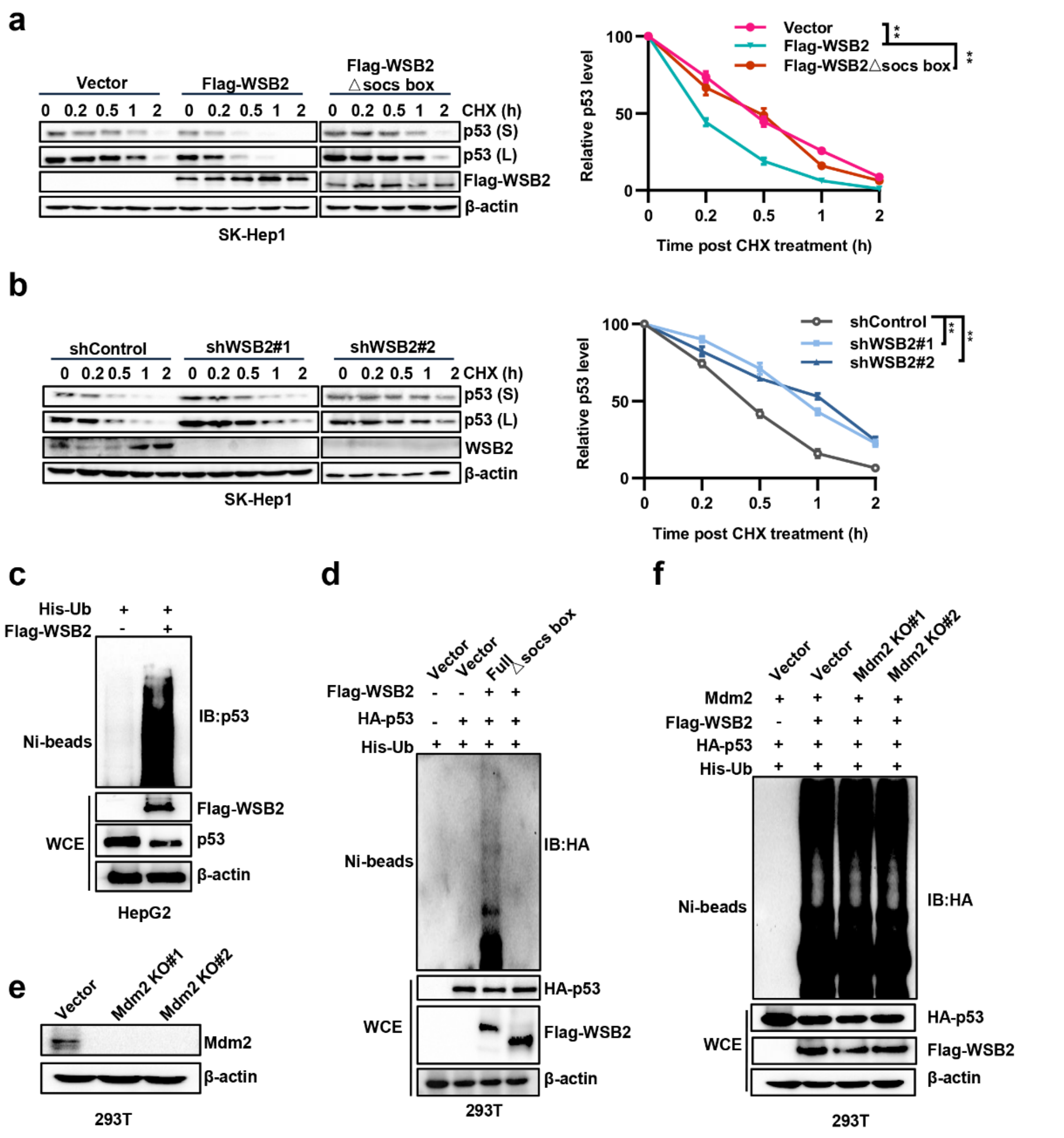

Supplementary Fig.7 WSB2-mediated p53 polyubiquitination is independent of Mdm2.

(a,b) Half-life of p53 controlled by WSB2 was analyzed by Western blotting. SK-Hep1 cells were transfected with Flag-WSB2 or its mutant ΔSOCS loss for 48 hours, or shRNA targeting WSB2 (shWSB2) for 48 hours and then exposed to 100 μg/ml cycloheximide (CHX) for 3 hours. The intensity of p53 expression for each band was normalized to β-actin. S, Short exposure; L, long exposure. **(c)** The *in vivo* ubiquitination assay indicated that WSB2 ubiquitinated wild-type p53 in HepG2 cells. HepG2 cells were transfected with the indicated plasmids for 48 hours, followed by MG132 (10 μM) treatment for 5 hours before lysis. **(d)** Full-length WSB2 but not its

ΔSOCS mutant ubiquitinates p53. HEK293T cells were transfected ubiquitin (Ub), with HA-p53, Flag-WSB2 or its ΔSOCS mutant for 48 hours, followed by MG132 treatment for 5 hours before lysis. **(e)** Western blotting analysis of the efficiency of Mdm2-KO in 293T cells. **(f)** The *in vivo* ubiquitination assays indicated that WSB2 ubiquitinated p53 in Mdm2- KO 293T cells. Mdm2-KO 293T cells were transfected with the indicated plasmids for 48 hours, followed by MG132 treatment for 5 hours before lysis. Data are shown as means ± SEMs. Statistical significance was calculated by two-way ANOVA with Bonferroni's multiple comparisons test. $^{**}p < 0.01$.

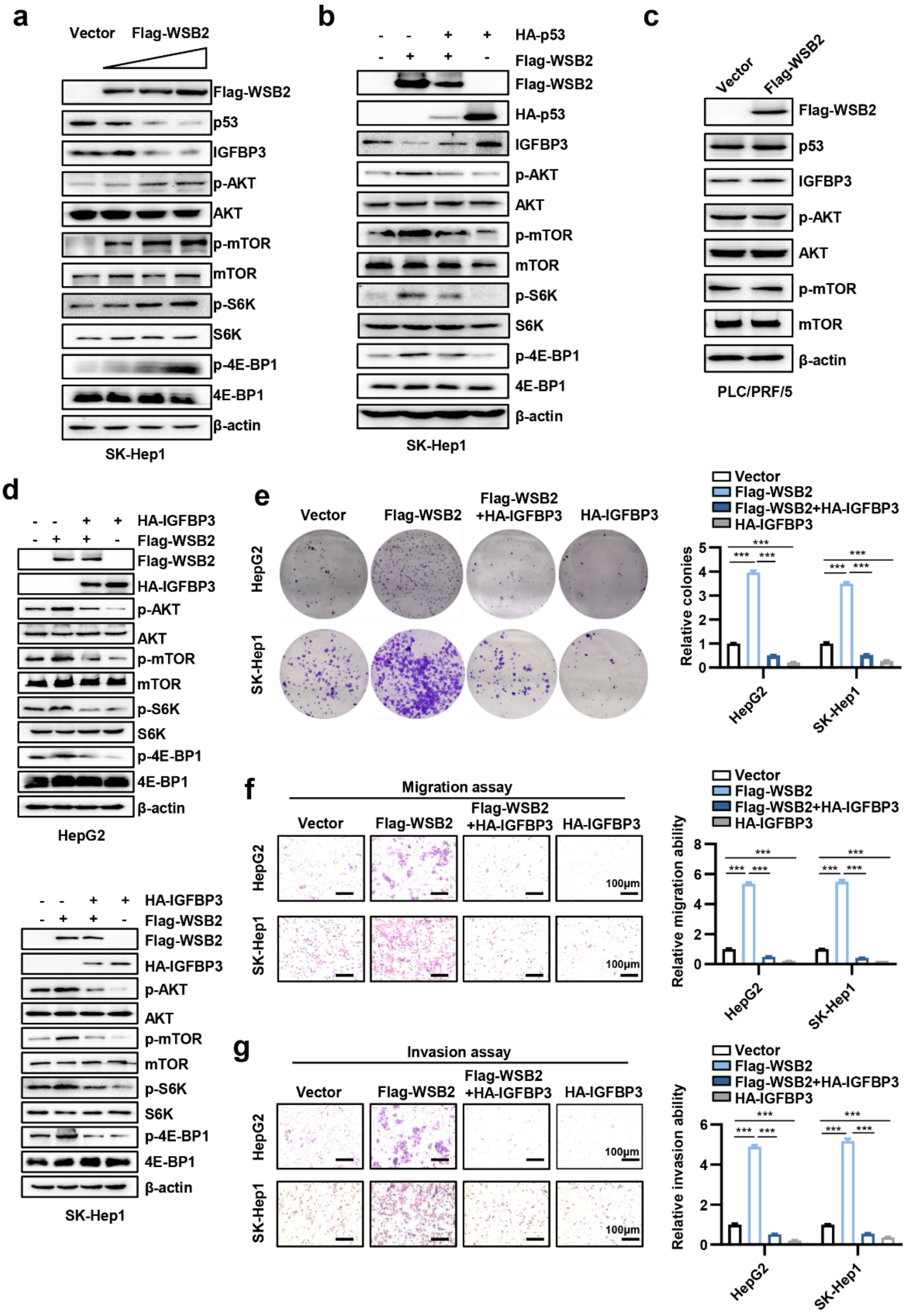

Supplementary Fig.8 p53 blocks WSB2-mediated the activation of IGFBP3-AKT-mTOR signaling pathway in p53 wild-type HCC cells

(a) WSB2 downregulated p53-IGFBP3 and upregulated AKT/mTOR activation in SK-Hep1 cells. SK-Hep1 cells were transfected with different amounts of Flag-WSB2 plasmids for 48 hours and then harvested for Western blotting with the indicated

antibodies. **(b)** WSB2 activated AKT/mTOR via degradation of p53. SK-Hep1 cells were transfected Flag-WSB2 plasmids with or without HA-p53 plasmids. After 48 hours transfection, cells were harvested and examined with the indicated antibodies. **(c)** WSB2 didn't regulate p53-IGFBP3-AKT-mTOR signaling pathway in TP53 mutant-type cells PLC/PRF/5. PLC/PRF/5 cells were transfected with indicated plasmids for 48 hours and then harvested for Western blotting with the indicated antibodies. **(d)** WSB2 activated AKT/mTOR via downregulation of IGFBP3. HepG2 or SK-Hep1 cells were transfected Flag-WSB2 plasmids with or without HA-IGFBP3 plasmids for 48 hours and then harvested for Western blotting with the indicated antibodies. **(e)** Representative images of cell colonies and quantification of colony numbers in WSB2-overexpressed cells with or without HA-IGFBP3 high expression were shown. n = 3. **(f,g)** Representative images and quantification of cell migration (f) and invasion (g) in WSB2-overexpressed cells with or without IGFBP3 overexpression were shown. n = 3. Scale bar, 100 μm. Data are shown as means ± SEMs. Statistical significance was calculated by One-way ANOVA with Tukey's multiple comparison test (e-g). $^{***}p < 0.001$.

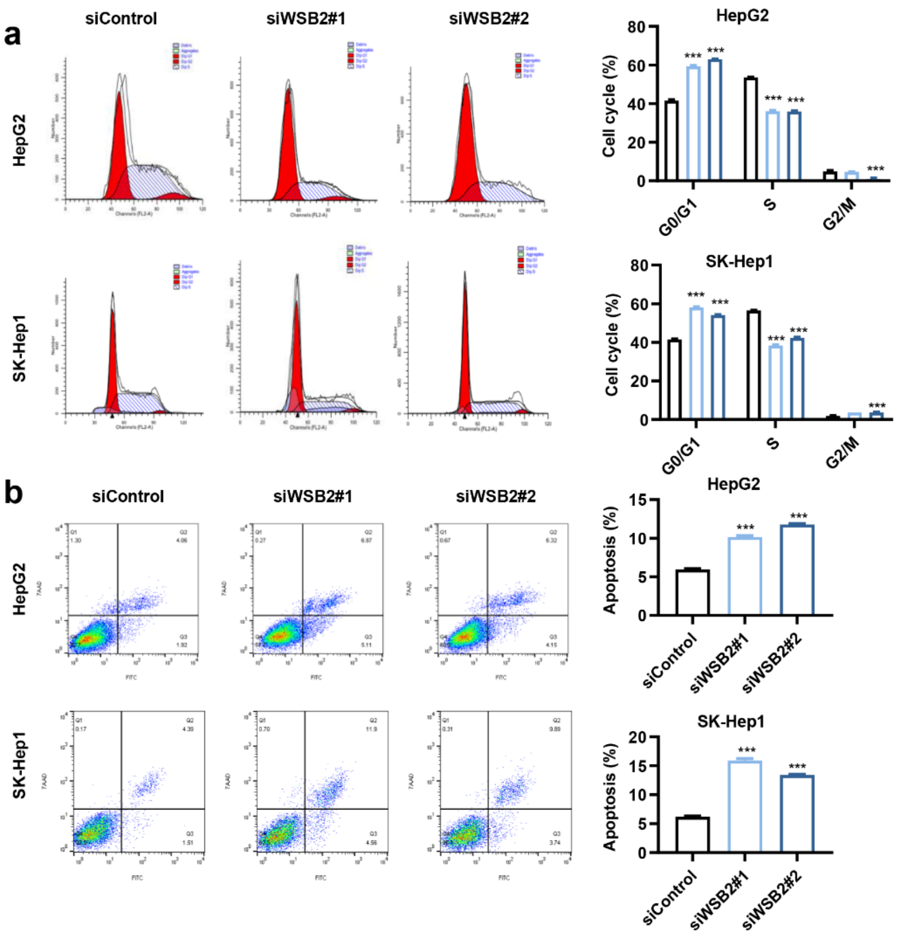

Supplementary Fig.9 Silencing of WSB2 induces cell cycle arrest at G1 phase and promotes apoptosis in p53 wild-type HCC cells.

(a) Silencing of WSB2 induced cell cycle arrest at G1 phase in p53 wild-type HCC cells. HepG2 or SK-Hep1 cells were transfected with siControl or siWSB2 for 48 hours and then stained with PI. Cell cycle was detected by flow cytometry. *n* = 3 **(b)** Silencing of WSB2 induces apoptosis in p53 wild-type HCC cells which were analyzed by flow cytometry. *n* = 3. Data are shown as means ± SEMs. The statistical significance was determined by Student's two-tailed t-test (a,b). ***$p < 0.001$.

Supplementary Table 1. 334 predicted E3 ligands which might ubiquinate p53

Gene Symbol (E3)	Gene Symbol (Substrate)	Domain_ Likeihood Ratio	Go_ Likelihood Ratio	Network_ Likelihood Ratio	Motif_ Likelihood Ratio	Confiden-ce Score	*p value*
RBBP6	TP53	12.12	3.03	3.83	9.23	0.957	NS
BAZ1B	TP53	12.12	2.76	3.83	9.23	0.956	0.023
TRIM33	TP53	12.12	5.94	3.83	4.28	0.956	NS
FBXO7	TP53	7.88	3.63	3.83	9.23	0.953	NS
MID2	TP53	7.88	3.63	3.83	9.23	0.953	NS
TBL1XR1	TP53	7.88	3.46	3.83	9.23	0.952	0.0015
AIRE	TP53	12.12	8.56	1.86	4.28	0.949	NS
MYCBP2	TP53	7.88	5.94	1.86	9.23	0.948	NS
TRAIP	TP53	7.88	2.78	3.83	9.23	0.947	0.00012
FBXW11	TP53	7.88	2.76	3.83	9.23	0.947	NS
RAPSN	TP53	7.88	2.76	3.83	9.23	0.947	NS
HECTD1	TP53	7.88	5.94	3.83	4.28	0.947	NS
UHRF2	TP53	12.12	3.63	1.86	9.23	0.947	NS
ZMYND11	TP53	12.12	3.03	1.86	9.23	0.943	NS
DZIP3	TP53	7.88	2.2	3.83	9.23	0.942	NS
FBXW2	TP53	7.88	2.2	3.83	9.23	0.942	NS
FBXW5	TP53	7.88	2.18	3.83	9.23	0.942	NS
ZMYND8	TP53	12.12	2.69	1.86	9.23	0.94	0.0065
HERC2	TP53	7.88	3.63	3.83	4.28	0.935	NS
TRIM21	TP53	7.88	3.63	3.83	4.28	0.935	0.0012
TRIM31	TP53	7.88	3.63	3.83	4.28	0.935	NS
TBL1X	TP53	7.88	3.46	1.86	9.23	0.935	NS
KAT2B	TP53	12.12	8.56	1	4.28	0.934	NS
FBXO31	TP53	7.88	5.94	1	9.23	0.933	NS
PML	TP53	7.88	5.85	1	9.23	0.933	0.0086
FBXL12	TP53	7.88	2.78	1.86	9.23	0.929	0.00047
RLIM	TP53	7.88	2.78	1.86	9.23	0.929	NS
PRPF19	TP53	12.12	5.94	3.83	1.32	0.928	＜0.0001
TRIM11	TP53	7.88	2.78	3.83	4.28	0.928	NS
TRIM22	TP53	7.88	2.78	3.83	4.28	0.928	NS
TRIP12	TP53	7.88	2.76	3.83	4.28	0.928	0.0099
RANBP2	TP53	12.12	3.46	1.86	4.28	0.926	NS
CREBBP	TP53	12.12	5.94	1	4.28	0.923	0.048
SALL2	TP53	1	8.56	3.83	9.23	0.923	NS
NEDD4	TP53	7.88	3.63	1.86	5.68	0.923	NS
KMT2C	TP53	12.12	2.69	1	9.23	0.923	NS
BRAP	TP53	7.88	2.2	1.86	9.23	0.922	0.00029
PJA1	TP53	7.88	2.2	1.86	9.23	0.922	NS
UBE4A	TP53	12.12	2.78	3.83	2.28	0.922	0.023

HERC3	TP53	7.88	2.2	3.83	4.28	0.921	NS
TRIM25	TP53	2.88	5.94	3.83	4.28	0.92	NS
FBXL2	TP53	7.88	3.63	1	9.23	0.918	0.049
MID1	TP53	7.88	3.63	1	9.23	0.918	0.0051
FUS	TP53	12.12	2.69	1.86	4.28	0.918	0.0034
AMFR	TP53	7.88	5.94	3.83	1.32	0.915	NS
RAE1	TP53	7.88	5.85	3.83	1.32	0.914	＜0.0001
TAF5	TP53	7.88	5.85	3.83	1.32	0.914	NS
TBL3	TP53	7.88	5.85	3.83	1.32	0.914	0.019
WWP2	TP53	7.88	2.78	1.86	5.68	0.914	0.02
FBXL17	TP53	7.88	3.07	1	9.23	0.913	NS
FBXL20	TP53	7.88	3.02	1	9.23	0.912	NS
FBXL22	TP53	7.88	3.02	1	9.23	0.912	NS
FBXO4	TP53	1	5.94	3.83	9.23	0.911	NS
CNOT4	TP53	1	5.85	3.83	9.23	0.91	0.0022
FBXO10	TP53	7.88	2.78	1	9.23	0.909	0.0013
FBXO17	TP53	7.88	2.78	1	9.23	0.909	NS
FBH1	TP53	7.88	2.78	1	9.23	0.909	NS
FBXO44	TP53	7.88	2.78	1	9.23	0.909	NS
FBXO6	TP53	7.88	2.78	1	9.23	0.909	0.024
FBXL5	TP53	7.88	2.78	1	9.23	0.909	NS
FBXL7	TP53	7.88	2.78	1	9.23	0.909	0.0012
FBXL8	TP53	7.88	2.78	1	9.23	0.909	NS
PJA2	TP53	7.88	2.78	1	9.23	0.909	NS
ZSWIM2	TP53	7.88	2.78	1	9.23	0.909	NS
FBXO45	TP53	7.88	2.76	1	9.23	0.909	0.012
FBXL3	TP53	7.88	2.76	1	9.23	0.909	NS
FBXL21P	TP53	7.88	2.76	1	9.23	0.909	NS
TRIM37	TP53	7.88	3.02	1.86	4.28	0.907	0.0079
TRIM63	TP53	7.88	3.02	1.86	4.28	0.907	NS
HERC1	TP53	7.88	2.78	1.86	4.28	0.904	NS
UBR5	TP53	7.88	2.76	1.86	4.28	0.904	＜0.0001
FBXO24	TP53	7.88	2.2	1	9.23	0.901	NS
FBXO3	TP53	7.88	2.2	1	9.23	0.901	NS
FBXO9	TP53	7.88	2.2	1	9.23	0.901	NS
FBXL14	TP53	7.88	2.2	1	9.23	0.901	NS
LTN1	TP53	7.88	2.2	1	9.23	0.901	NS
SCAF11	TP53	7.88	2.2	1	9.23	0.901	NS
TTC3	TP53	7.88	2.2	1	9.23	0.901	NS
ZNRF1	TP53	7.88	2.2	1	9.23	0.901	NS
ZNRF2	TP53	7.88	2.2	1	9.23	0.901	NS
KAT2A	TP53	12.12	3.03	1	4.28	0.9	0.023
FBXW10	TP53	7.88	1.99	1	9.23	0.897	NS
FBXW12	TP53	7.88	1.99	1	9.23	0.897	NS

FBXO15	TP53	7.88	1.99	1	9.23	0.897	NS
FBXO33	TP53	7.88	1.99	1	9.23	0.897	NS
FBXO39	TP53	7.88	1.99	1	9.23	0.897	NS
FBXO41	TP53	7.88	1.99	1	9.23	0.897	0.019
FBXO48	TP53	7.88	1.99	1	9.23	0.897	NS
FBXO8	TP53	7.88	1.99	1	9.23	0.897	NS
FBXW4	TP53	7.88	1.99	1	9.23	0.897	NS
FBXW8	TP53	7.88	1.99	1	9.23	0.897	NS
FBXW9	TP53	7.88	1.99	1	9.23	0.897	0.0086
WDR12	TP53	7.88	3.63	3.83	1.32	0.897	＜0.0001
UBOX5	TP53	12.12	2.69	1.86	2.28	0.895	NS
HERC5	TP53	7.88	2.2	1.86	4.28	0.895	NS
TRIM62	TP53	7.88	2.2	1.86	4.28	0.895	0.0015
RNF139	TP53	7.88	3.46	3.83	1.32	0.895	NS
SMURF1	TP53	3.9	5.94	1	5.68	0.893	NS
SYTL4	TP53	3.9	3.63	1	9.23	0.892	NS
SMURF2	TP53	3.9	5.85	1	5.68	0.892	0.012
ARIH1	TP53	1	3.63	3.83	9.23	0.892	NS
BFAR	TP53	1	3.63	3.83	9.23	0.892	NS
FANCL	TP53	1	3.63	3.83	9.23	0.892	＜0.0001
RAG1	TP53	1	3.63	3.83	9.23	0.892	NS
SHPRH	TP53	1	3.63	3.83	9.23	0.892	NS
ZBTB11	TP53	1	3.63	3.83	9.23	0.892	NS
TRIM17	TP53	7.88	3.63	1	4.28	0.89	NS
TRIM26	TP53	7.88	3.63	1	4.28	0.89	NS
TRIM27	TP53	7.88	3.63	1	4.28	0.89	NS
TRIM38	TP53	7.88	3.63	1	4.28	0.89	0.049
TRIM41	TP53	7.88	3.63	1	4.28	0.89	0.027
TRIM50	TP53	7.88	3.63	1	4.28	0.89	NS
TRIM4	TP53	7.88	3.63	1	4.28	0.89	NS
TRIM5	TP53	7.88	3.63	1	4.28	0.89	NS
TRIM6	TP53	7.88	3.63	1	4.28	0.89	0.02
TRIM7	TP53	7.88	3.63	1	4.28	0.89	0.0097
HLTF	TP53	1	3.46	3.83	9.23	0.89	0.044
BRWD1	TP53	12.12	1.99	3.83	1.32	0.89	NS
KATNB1	TP53	7.88	3.02	3.83	1.32	0.889	0.0016
DCAF7	TP53	7.88	2.78	3.83	1.32	0.885	0.00013
GNB3	TP53	7.88	2.78	3.83	1.32	0.885	NS
RNF130	TP53	7.88	2.78	3.83	1.32	0.885	NS
NLE1	TP53	7.88	2.78	3.83	1.32	0.885	＜0.0001
PLAA	TP53	7.88	2.78	3.83	1.32	0.885	NS
RNF183	TP53	7.88	2.78	3.83	1.32	0.885	NS
WDR47	TP53	7.88	2.78	3.83	1.32	0.885	NS
WDR5	TP53	7.88	2.78	3.83	1.32	0.885	0.029

WDR61	TP53	7.88	2.78	3.83	1.32	0.885	NS
CIAO1	TP53	7.88	2.76	3.83	1.32	0.885	0.00062
PAFAH1B1	TP53	7.88	2.76	3.83	1.32	0.885	NS
SEC13	TP53	7.88	2.76	3.83	1.32	0.885	0.00047
TLE2	TP53	7.88	2.76	3.83	1.32	0.885	NS
FBXO5	TP53	1	3.07	3.83	9.23	0.884	＜0.0001
PRC1	TP53	1	3.02	3.83	9.23	0.884	NS
SALL1	TP53	1	5.94	1.86	9.23	0.882	NS
ZMIZ1	TP53	1	5.94	1.86	9.23	0.882	NS
TRIM54	TP53	7.88	3.02	1	4.28	0.882	＜0.0001
PRKN	TP53	1	5.85	1.86	9.23	0.881	NS
CCNF	TP53	1	2.78	3.83	9.23	0.88	＜0.0001
FBXL18	TP53	1	2.78	3.83	9.23	0.88	＜0.0001
IRF2BP1	TP53	1	2.78	3.83	9.23	0.88	NS
G2E3	TP53	7.88	2.78	1	4.28	0.878	NS
HACE1	TP53	7.88	2.78	1	4.28	0.878	0.00049
AREL1	TP53	7.88	2.78	1	4.28	0.878	NS
TRIM13	TP53	7.88	2.78	1	4.28	0.878	NS
TRIM35	TP53	7.88	2.78	1	4.28	0.878	NS
TRIM36	TP53	7.88	2.78	1	4.28	0.878	0.021
HERC4	TP53	7.88	2.76	1	4.28	0.877	NS
TRIM71	TP53	7.88	2.76	1	4.28	0.877	NS
TRIM46	TP53	7.88	2.76	1	4.28	0.877	0.049
TRIM67	TP53	7.88	2.76	1	4.28	0.877	NS
HECW1	TP53	3.9	2.2	1.86	5.68	0.876	0.00094
HECW2	TP53	3.9	2.2	1.86	5.68	0.876	NS
WDR77	TP53	7.88	2.2	3.83	1.32	0.875	＜0.0001
GNB2	TP53	7.88	2.18	3.83	1.32	0.874	0.0024
NFX1	TP53	1	8.56	1	9.23	0.87	NS
CCNB1IP1	TP53	1	2.2	3.83	9.23	0.869	NS
LRSAM1	TP53	1	2.2	3.83	9.23	0.869	NS
RBSN	TP53	1	2.2	3.83	9.23	0.869	NS
RMND5A	TP53	1	2.2	3.83	9.23	0.869	NS
TRPC4AP	TP53	1	2.18	3.83	9.23	0.868	＜0.0001
ITCH	TP53	3.9	3.46	1	5.68	0.868	NS
PRPF4	TP53	7.88	1.92	3.83	1.32	0.868	0.027
STRN4	TP53	7.88	1.92	3.83	1.32	0.868	＜0.0001
MARCHF7	TP53	1	5.94	3.83	3.35	0.868	NS
HECTD2	TP53	7.88	2.2	1	4.28	0.867	NS
HECTD3	TP53	7.88	2.2	1	4.28	0.867	＜0.0001
HERC6	TP53	7.88	2.2	1	4.28	0.867	0.014
TRIM49B	TP53	7.88	2.2	1	4.28	0.867	NS
TRIM10	TP53	7.88	2.2	1	4.28	0.867	NS
TRIM15	TP53	7.88	2.2	1	4.28	0.867	NS

TRIM34	TP53	7.88	2.2	1	4.28	0.867	0.0074
TRIM45	TP53	7.88	2.2	1	4.28	0.867	0.0014
TRIM48	TP53	7.88	2.2	1	4.28	0.867	NS
TRIM49	TP53	7.88	2.2	1	4.28	0.867	NS
TRIM52	TP53	7.88	2.2	1	4.28	0.867	0.016
TRIM55	TP53	7.88	2.2	1	4.28	0.867	NS
TRIM56	TP53	7.88	2.2	1	4.28	0.867	NS
TRIM58	TP53	7.88	2.2	1	4.28	0.867	NS
TRIM60	TP53	7.88	2.2	1	4.28	0.867	NS
TRIM61	TP53	7.88	2.2	1	4.28	0.867	0.0094
TRIM65	TP53	7.88	2.2	1	4.28	0.867	<0.0001
TRIM68	TP53	7.88	2.2	1	4.28	0.867	NS
TRIM72	TP53	7.88	2.2	1	4.28	0.867	NS
TRIM73	TP53	7.88	2.2	1	4.28	0.867	NS
TRIM74	TP53	7.88	2.2	1	4.28	0.867	NS
TRIM2	TP53	7.88	2.2	1	4.28	0.867	NS
TRIM9	TP53	7.88	2.2	1	4.28	0.867	NS
UBE3C	TP53	7.88	2.2	1	4.28	0.867	NS
ECT2L	TP53	7.88	1	1	9.23	0.865	NS
FBXO16	TP53	7.88	1	1	9.23	0.865	0.0068
FBXO46	TP53	7.88	1	1	9.23	0.865	0.00087
CDCA3	TP53	1	1.99	3.83	9.23	0.864	<0.0001
CDC20	TP53	7.88	3.63	1.86	1.32	0.864	<0.0001
DDB2	TP53	7.88	3.63	1.86	1.32	0.864	NS
RNF11	TP53	7.88	3.63	1.86	1.32	0.864	0.03
UBE3B	TP53	7.88	1.99	1	4.28	0.861	0.029
WSB2	TP53	7.88	1.99	1	4.28	0.861	<0.0001
EED	TP53	7.88	3.46	1.86	1.32	0.861	<0.0001
ZNRF3	TP53	7.88	2.2	3.83	1	0.861	NS
PHF21A	TP53	12.12	2.78	1.86	1	0.858	0.0012
CORO7	TP53	7.88	1.84	1	4.28	0.857	NS
GEMIN5	TP53	7.88	5.94	1	1.32	0.857	0.025
NUP43	TP53	7.88	5.94	1	1.32	0.857	<0.0001
RNF103	TP53	7.88	5.94	1	1.32	0.857	NS
SEH1L	TP53	7.88	5.94	1	1.32	0.857	NS
THOC6	TP53	7.88	5.94	1	1.32	0.857	0.023
DCAF13	TP53	7.88	5.85	1	1.32	0.856	<0.0001
GNB1	TP53	7.88	5.85	1	1.32	0.856	<0.0001
RACK1	TP53	7.88	5.85	1	1.32	0.856	-
PLRG1	TP53	7.88	5.85	1	1.32	0.856	0.0022
PWP2	TP53	7.88	5.85	1	1.32	0.856	0.003
RBBP4	TP53	7.88	5.85	1	1.32	0.856	0.00029
RBBP7	TP53	7.88	5.85	1	1.32	0.856	<0.0001
WDSUB1	TP53	12.12	2.2	1	2.28	0.856	NS

PHC1	TP53	1	3.63	3.83	4.28	0.855	NS
RABGEF1	TP53	1	3.63	3.83	4.28	0.855	0.011
SOCS3	TP53	1	3.63	3.83	4.28	0.855	NS
SOCS5	TP53	1	3.63	3.83	4.28	0.855	NS
SOCS7	TP53	1	3.63	3.83	4.28	0.855	0.015
BMI1	TP53	1	3.46	1.86	9.23	0.855	＜0.0001
LITAF	TP53	1	3.46	1.86	9.23	0.855	NS
APAF1	TP53	7.88	3.07	1.86	1.32	0.855	0.032
RNF111	TP53	7.88	3.02	1.86	1.32	0.854	NS
RNF4	TP53	7.88	3.02	1.86	1.32	0.854	0.022
MALT1	TP53	1	3.46	3.83	4.28	0.852	NS
TEP1	TP53	7.88	5.35	1	1.32	0.851	NS
WDR3	TP53	7.88	5.35	1	1.32	0.851	0.00026
MNAT1	TP53	1	5.94	1	9.23	0.851	0.019
MGRN1	TP53	1	5.94	1	9.23	0.851	NS
NEURL1	TP53	1	5.94	1	9.23	0.851	-
ZMIZ2	TP53	1	5.94	1	9.23	0.851	0.00043
BARD1	TP53	1	5.85	1	9.23	0.85	NS
OSTM1	TP53	1	5.85	1	9.23	0.85	0.00056
RC3H1	TP53	1	5.85	1	9.23	0.85	NS
FZR1	TP53	7.88	2.78	1.86	1.32	0.849	NS
RBBP5	TP53	7.88	2.76	1.86	1.32	0.849	NS
TLE1	TP53	7.88	2.76	1.86	1.32	0.849	0.012
CHFR	TP53	1	3.02	1.86	9.23	0.847	0.0051
BABAM2	TP53	1	3.03	3.83	4.28	0.845	-
NSMCE1	TP53	1	5.35	1	9.23	0.845	0.022
NSMCE2	TP53	1	5.35	1	9.23	0.845	0.01
RBCK1	TP53	1	2.78	1.86	9.23	0.843	0.015
RING1	TP53	1	2.78	1.86	9.23	0.843	NS
KDM2A	TP53	1	2.76	1.86	9.23	0.842	NS
RBX1	TP53	1	2.76	1.86	9.23	0.842	＜0.0001
FANCC	TP53	1	5.94	1.86	4.28	0.842	NS
TMF1	TP53	1	5.85	1.86	4.28	0.841	NS
ELOA	TP53	1	2.78	3.83	4.28	0.84	-
IRAK4	TP53	1	2.78	3.83	4.28	0.84	NS
RNF144A	TP53	1	2.78	3.83	4.28	0.84	0.0018
RWDD3	TP53	1	2.78	3.83	4.28	0.84	NS
TRIM32	TP53	1	2.78	3.83	4.28	0.84	0.0055
TRIML1	TP53	2.88	3.63	1	4.28	0.839	-
TRIML2	TP53	2.88	3.63	1	4.28	0.839	-
BCL6B	TP53	1	8.56	3.83	1.32	0.837	NS
BUB3	TP53	7.88	2.2	1.86	1.32	0.836	＜0.0001
CSTF1	TP53	7.88	2.2	1.86	1.32	0.836	NS
DCAF6	TP53	7.88	2.2	1.86	1.32	0.836	NS

RNF10	TP53	7.88	2.2	1.86	1.32	0.836	0.0071
EIF3I	TP53	7.88	2.18	1.86	1.32	0.835	0.0035
WDR37	TP53	7.88	1	3.83	1.32	0.832	0.034
FBXO30	TP53	1	2.2	1.86	9.23	0.829	＜0.0001
XIAP	TP53	1	2.2	1.86	9.23	0.829	NS
PEX7	TP53	7.88	3.63	1	1.32	0.829	NS
RNF6	TP53	7.88	3.63	1	1.32	0.829	NS
WDTC1	TP53	7.88	3.63	1	1.32	0.829	0.022
GTF3C2	TP53	7.88	1.92	1.86	1.32	0.828	＜0.0001
HSPA8	TP53	1	8.56	1	4.28	0.827	0.0023
CKS1B	TP53	1	2.2	3.83	4.28	0.826	0.00039
NEURL2	TP53	1	2.2	3.83	4.28	0.826	NS
ZFP91	TP53	1	2.2	3.83	4.28	0.826	NS
RNF207	TP53	7.88	3.46	1	1.32	0.826	NS
BTBD1	TP53	1	2.18	3.83	4.28	0.825	NS
FBXO28	TP53	1	1	3.83	9.23	0.825	NS
SPSB4	TP53	2.88	2.76	1	4.28	0.822	0.021
PHF14	TP53	12.12	2.78	1	1	0.822	0.036
PHRF1	TP53	12.12	2.78	1	1	0.822	0.02
FBXO21	TP53	1	3.63	1	9.23	0.821	NS
MKRN3	TP53	1	3.63	1	9.23	0.821	0.01
RC3H2	TP53	1	3.63	1	9.23	0.821	NS
UNKL	TP53	1	3.63	1	9.23	0.821	0.0082
PCGF6	TP53	1	8.56	3.83	1	0.82	NS
ASB13	TP53	1	1.99	3.83	4.28	0.82	NS
ASB2	TP53	1	1.99	3.83	4.28	0.82	NS
FANCE	TP53	1	1.99	3.83	4.28	0.82	＜0.0001
FANCF	TP53	1	1.99	3.83	4.28	0.82	0.012
SOCS6	TP53	1	1.99	3.83	4.28	0.82	NS
GZF1	TP53	1	3.46	1	9.23	0.818	0.00013
PHIP	TP53	12.12	1.99	1	1.32	0.818	NS
BIRC7	TP53	1	3.63	3.83	2.28	0.818	NS
KEAP1	TP53	1	3.63	3.83	2.28	0.818	0.021
GRWD1	TP53	7.88	3.03	1	1.32	0.817	＜0.0001
PWP1	TP53	7.88	3.03	1	1.32	0.817	＜0.0001
GNB5	TP53	7.88	3.02	1	1.32	0.817	NS
POC1A	TP53	7.88	3.02	1	1.32	0.817	0.028
POC1B	TP53	7.88	3.02	1	1.32	0.817	NS
RNF32	TP53	7.88	3.02	1	1.32	0.817	NS
TAF5L	TP53	7.88	3.02	1	1.32	0.817	NS
PAAF1	TP53	7.88	2.78	1	1.32	0.812	0.035
RNF115	TP53	7.88	2.78	1	1.32	0.812	NS
RNF149	TP53	7.88	2.78	1	1.32	0.812	NS
RNF43	TP53	7.88	2.78	1	1.32	0.812	NS

SMU1	TP53	7.88	2.78	1	1.32	0.812	0.0096
EIPR1	TP53	7.88	2.78	1	1.32	0.812	-
WDHD1	TP53	7.88	2.78	1	1.32	0.812	0.005
WDR18	TP53	7.88	2.78	1	1.32	0.812	NS
WDR48	TP53	7.88	2.78	1	1.32	0.812	0.029
WDR6	TP53	7.88	2.78	1	1.32	0.812	0.00026
WDR92	TP53	7.88	2.78	1	1.32	0.812	0.028
DCUN1D1	TP53	1	3.63	1.86	4.28	0.812	NS
RMND5B	TP53	1	3.63	1.86	4.28	0.812	0.017
UBE2D1	TP53	1	3.63	1.86	4.28	0.812	0.018
VHL	TP53	1	3.63	1.86	4.28	0.812	0.0055
AAMP	TP53	7.88	2.76	1	1.32	0.811	0.0029
ERCC8	TP53	7.88	2.76	1	1.32	0.811	NS
MLST8	TP53	7.88	2.76	1	1.32	0.811	0.0014
TLE3	TP53	7.88	2.76	1	1.32	0.811	NS
WDR1	TP53	7.88	2.76	1	1.32	0.811	0.0016
WDR41	TP53	7.88	2.76	1	1.32	0.811	0.0041
TBL1Y	TP53	7.88	2.69	1	1.32	0.81	NS
WDR82	TP53	7.88	2.69	1	1.32	0.81	NS
KDM1B	TP53	1	3.03	1	9.23	0.809	NS
ZEB2	TP53	1	3.03	1	9.23	0.809	NS
FBXO32	TP53	1	3.02	1	9.23	0.809	NS
AURKB	TP53	1	3.46	1.86	4.28	0.808	0.00045
CBX4	TP53	1	3.46	1.86	4.28	0.808	NS
MED8	TP53	1	3.46	1.86	4.28	0.808	＜0.0001
SPSB1	TP53	2.88	2.2	1	4.28	0.807	NS
SPSB2	TP53	2.88	2.2	1	4.28	0.807	0.00035
TRIM64B	TP53	2.88	2.2	1	4.28	0.807	-
TRIM64C	TP53	2.88	2.2	1	4.28	0.807	-
TRIM43	TP53	2.88	2.2	1	4.28	0.807	-
KLHL20	TP53	1	3.02	3.83	2.28	0.806	NS

Supplementary Table 2. Relationships between WSB2 and clinicopathological parameters in the IHC cohort (n=113)

		WSB2			
**Characteristics**	**NO. patients**	**Low**	**High**	χ^2	***p* value**
Age (years)					
≤60	96	36	60	6.471	0.011
＞60	17	12	5		
Gender					
Male	100	44	56	0.824	0.364
Female	13	4	9		
Tumor size (cm)					
＜5cm	30	21	9	12.662	＜0.001
≥5cm	83	27	56		
Tumor multiplicity					
Single	89	40	49	1.043	0.307
Multiple	24	8	16		
Lymph node metastasis					
Negative	110	48	62	2.276	0.131
Positive	3	0	3		
Distant metastasis					
Negative	73	36	37	3.945	0.047
Positive	40	12	28		
TNM stage					
I+II	51	32	19	15.626	＜0.001
III+IV	62	16	46		
Differentiation					
Well-moderate	95	42	53	0.733	0.392
Poor-undifferentiation	18	6	12		

Vascular invasion					
Negative	80	39	41	4.410	0.036
Positive	33	9	24		

Supplementary Table 3. Cox univariate and multivariate analysis of predictors of overall survival following hepatectomy in the IHC cohort (n=113)

Variables for overall survival	Univariate analysis		Multivariate analysis	
	Hazard ratio/ CI（95%）	*p* value	Hazard ratio/ CI（95%）	*p* value
Gender				
(female versus male)	1.404/0.676-2.916	0.363		
Age, year				
(＞60 versus ≤60)	0.611/0.305-1.224	0.165		
Tumor multiplicity				
(multiple versus single)	1.675/1.013-2.768	0.044	NA	NA
Tumor differentiation				
(well-moderate versus poor-undifferentiation)	0.557/0.316-0.985	0.044	NA	NA
Lymph node metastasis				
(positive versus negative)	1.178/0.289-4.800	0.820		
Distant metastasis				
(positive versus negative)	2.060/1.314-3.229	0.002	NA	NA
Tumor size, cm				
(≥5 versus ＜5)	2.585/1.467-4.552	0.001	NA	NA
Vascular invasion				
(positive versus negative)	2.670/1.667-4.275	＜0.001	NA	NA
TNM stage				
(III+IV versus I+II)	3.028/1.881-4.875	＜0.001	2.527/1.538-4.153	＜0.001
WSB2 expression level				
(low versus high)	0.412/0.259-0.656	＜0.001	0.535/0.330-0.869	0.011

Supplementary Table 4. Cox univariate and multivariate analysis of predictors of recurrence in HCC patients following hepatectomy in the IHC cohort (n=113)

Variables for tumor recurrence	Univariate analysis		Multivariate analysis	
	Hazard ratio/ CI（95%）	*p* value	Hazard ratio/ CI（95%）	*p* value
Gender				
(female versus male)	1.380/0.665-2.863	0.387		
Age, year				
(＞60 versus ≤60)	0.485/0.242-0.972	0.041	NA	NA
Tumor multiplicity				
(multiple versus single)	1.639/0.968-2.777	0.066		
Tumor differentiation				
(well-moderate versus poor-undifferentiation)	0.732/0.404-1.328	0.305		
Lymph node metastasis				
(positive versus negative)	1.119/0.274-4.565	0.876		
Distant metastasis				
(positive versus negative)	2.502/1.608-3.893	＜0.001	1.742/1.078-2.813	0.023
Tumor size, cm				
(≥5 versus ＜5)	1.863/1.133-3.065	0.014	NA	NA
Vascular invasion				
(positive versus negative)	3.031/1.865-4.926	＜0.001	NA	NA
TNM stage				
(III+IV versus I+II)	3.217/2.028-5.104	＜0.001	2.385/1.427-3.987	0.001
WSB2 expression level				
(low versus high)	0.440/0.281-0.690	＜0.001	0.611/0.377-0.991	0.046

Supplementary Table 5. Relationships between WSB2 and clinicopathological parameters in TP53 wild-type HCC patients (n=79)

		**WSB2**			
**Characteristics**	**NO. patients**	**Low**	**High**	χ^2	***p* value**
Age (years)					
≤60	71	29	42	1.375	0.241
＞60	8	5	3		
Gender					
Male	73	32	41	0.249	0.617
Female	6	2	4		
Tumor size (cm)					
＜5cm	20	15	5	11.159	0.001
≥5cm	59	19	40		
Tumor multiplicity					
Single	60	27	33	0.392	0.531
Multiple	19	7	12		
Lymph node metastasis					
Negative	77	34	43	1.550	0.213
Positive	2	0	2		
Distant metastasis					
Negative	51	27	24	5.756	0.016
Positive	28	7	21		
TNM stage					
I+II	34	25	9	22.637	＜0.001
III+IV	45	9	36		
Differentiation					
Well-moderate	65	30	35	1.453	0.228
Poor-undifferentiation	14	4	10		

Vascular invasion					
Negative	55	30	25	9.779	0.002
Positive	24	4	20		

Supplementary Table 6. Relationships between WSB2 and clinicopathological parameters in TP53 mutated HCC patients (n=34)

		WSB2			
**Characteristics**	**NO. patients**	**Low**	**High**	χ^2	***p* value**
Age (years)					
≤60	25	7	18	6.770	0.009
＞60	9	7	2		
Gender					
Male	7	2	5	0.578	0.447
Female	27	12	15		
Tumor size (cm)					
＜5cm	10	6	4	2.072	0.150
≥5cm	24	8	16		
Tumor multiplicity					
Single	29	13	16	1.085	0.298
Multiple	5	1	4		
Lymph node metastasis					
Negative	33	14	19	0.721	0.396
Positive	1	0	1		
Distant metastasis					
Negative	22	9	13	0.002	0.966
Positive	12	5	7		
TNM stage					
I+II	17	7	10	0.000	1.000
III+IV	17	7	10		
Differentiation					
Well-moderate	30	12	18	0.146	0.703
Poor-undifferentiation	4	2	2		

Vascular invasion					
Negative	25	9	16	1.045	0.307
Positive	9	5	4		

Supplementary Table 7. Summary of mass spectrometry (MS) analysis of WSB2 interactors in HEK-293T

Identified proteins	Unique Peptides	Protein Accession	Protein Description
WSB2	11	F5H280	WD repeat and SOCS box-containing protein 2
TUBB2C	3	Q8N6N5	Tubulin beta chain
HSPA8	1	E9PN89	Heat shock cognate 71 kDa protein
TUBB2A	1	Q13885	Tubulin beta-2A chain
TUBB6	4	Q9BUF5	Tubulin beta-6 chain
CKB	6	A0A0S2Z471	Creatine kinase (Fragment)
CLTC	7	A0A087WVQ6	Clathrin heavy chain
XRCC5	7	P13010	X-ray repair cross-complementing protein 5
TP53	4	L0EQ92	Tumor suppressor p53
H3C1	1	P68431	Histone H3.1
BAG2	7	O95816	BAG family molecular chaperone regulator 2
SLC25A6	1	P12236	ADP/ATP translocase 3
EIF5A	2	P63241	Eukaryotic translation initiation factor 5A-1
SUPT16H	4	Q9Y5B9	FACT complex subunit SPT16
PSMB5	5	P28074	Proteasome subunit beta type-5
HSPH1	4	A0A024RDQ0	Heat shock 110 kDa protein
HNRNPH2	1	P55795	Heterogeneous nuclear ribonucleoprotein H2
NONO	2	A0A7I2V464	Non-POU domain-containing octamer-binding protein
MYH10	5	P35580	Myosin-10
RPS23	2	P62266	40S ribosomal protein S23
PFN1	3	P07737	Profilin-1

BAG6	5	A0A024RCR6	BCL2-associated athanogene 6
SRM	1	K7ESL0	Spermidine synthase
GNB3	1	F5H0S8	Guanine nucleotide-binding protein G(I)/G(S)/G(T) subunit beta-3
HPRT1	1	P00492	Hypoxanthine-guanine phosphoribosyltransferase
FEN1	1	F5H1Y3	Flap endonuclease 1
SLC25A1	2	D9HTE9	Plasma membrane citrate carrier
PDIA3	2	H7BZJ3	Protein disulfide-isomerase A3
PSMB4	2	P28070	Proteasome subunit beta type-4
SHINC3	2	A6QKW0	SHINC3
PSMB6	2	P28072	Proteasome subunit beta type-6
AIFM1	2	O95831	Apoptosis-inducing factor 1, mitochondrial
PSMD14	2	O00487	26S proteasome non-ATPase regulatory subunit 14
EIF2B1	1	H0YGG4	Translation initiation factor eIF-2B subunit alpha
PRPF19	2	Q9UMS4	Pre-mRNA-processing factor 19
HRMT1L2	1	Q5U8W9	Protein arginine methyltransferase 1 isoform 4
UQCRFS1P1	1	P0C7P4	Putative cytochrome b-c1 complex subunit Rieske-like protein 1
RUVBL2	2	Q9Y230	RuvB-like 2
KCTD2	2	Q8IYY2	KCTD2 protein
PFDN1	2	E5RGS4	Prefoldin subunit 1
KPNB1	2	Q14974	Importin subunit beta-1
SDHA	1	A0A087X1I3	Flavoprotein subunit of complex II
MCM7	2	P33993	DNA replication licensing factor MCM7

CLU	1	Q8IWM0	Clusterin (Fragment)
RPL35	1	P42766	60S ribosomal protein L35
ARHGDIA	1	P52565	Rho GDP-dissociation inhibitor 1
ARF4	1	C9JPM4	ADP-ribosylation factor 4
SFXN1	1	D6RFI0	Sideroflexin-1
NTPCR	2	Q5TDF0	Cancer-related nucleoside-triphosphatase
DKFZp686G2045	1	Q68E00	Uncharacterized protein DKFZp686G2045
UMPS	2	P11172	Uridine 5'-monophosphate synthase
HAX1	1	E9PIQ7	HCLS1-associated protein X-1
CTPS1	1	B4E1E0	CTP synthase
HACD3	1	Q9P035	Very-long-chain (3R)-3-hydroxyacyl-CoA dehydratase 3
ANXA5	1	E9PHT9	Annexin
PSMB2	6	P49721	Proteasome subunit beta type-2
PTMAP7	2	Q53S24	Prothymosin, alpha
DNAJA1	4	P31689	DnaJ homolog subfamily A member 1
RAN	4	B5MDF5	GTP-binding nuclear protein Ran
HNRNPF	3	P52597	Heterogeneous nuclear ribonucleoprotein F
EPRS1	3	P07814	Bifunctional glutamate/proline--tRNA ligase
GNAI2	2	P04899	Guanine nucleotide-binding protein G(i) subunit alpha-2
DDB1	5	Q16531	DNA damage-binding protein 1
HSPE1	2	A0A024R3X7	Heat shock 10kDa protein 1 (Chaperonin 10), isoform CRA_d
PSMC6	4	A0A087X2I1	26S proteasome regulatory subunit 10B
ELOC	4	Q15369	Elongin-C

GART	2	Q3B7A7	Trifunctional purine biosynthetic protein adenosine-3
DNAJA2	3	O60884	DnaJ homolog subfamily A member 2
PSMC4	5	P43686	26S proteasome regulatory subunit 6B
PSMD12	2	O00232	26S proteasome non-ATPase regulatory subunit 12
CFL1	2	E9PK25	Cofilin, non-muscle isoform
PLEC	5	Q15149	Plectin
PSMD1	2	Q99460	26S proteasome non-ATPase regulatory subunit 1
PGK1	3	P00558	Phosphoglycerate kinase 1
VBP1	3	P61758	Prefoldin subunit 3
ERLIN1	2	O75477	Erlin-1
PFDN2	1	Q9UHV9	Prefoldin subunit 2
CUL5	2	Q93034	Cullin-5
MAGED2	3	Q5H909	Melanoma-associated antigen D2
YWHAH	1	Q04917	14-3-3 protein eta
PDIA6	1	Q15084	Protein disulfide-isomerase A6
PFDN5	2	F8VNW6	Prefoldin subunit 5
DUT	1	H0YNJ9	Deoxyuridine 5'-triphosphate nucleotidohydrolase
SEC61A1	1	B4DR61	Protein transport protein Sec61 subunit alpha isoform 1
ARL1	1	B4DZG7	ADP-ribosylation factor-like protein 1
PRDX6	1	P30041	Peroxiredoxin-6
AGPS	1	A0A7P0TAU9	Alkylglycerone-phosphate synthase
SF3B3	1	Q15393	Splicing factor 3B subunit 3
FARSA	1	K7ER16	Phenylalanine--tRNA ligase alpha subunit

RPS12	1	P25398	40S ribosomal protein S12
MYL6	1	B7Z6Z4	Myosin light polypeptide 6
RBBP7	1	E9PC52	Histone-binding protein RBBP7
PSMB7	2	Q99436	Proteasome subunit beta type-7
CYCS	1	C9JFR7	Cytochrome c
DNAJB2	1	C9JRD2	DnaJ homolog subfamily B member 2
SET	1	Q01105	Protein SET
SMG7	1	E9PD50	Protein SMG7
DDOST	1	P39656	Dolichyl-diphosphooligosaccharide--protein glycosyltransferase 48 kDa subunit
SDHB	1	P21912	Succinate dehydrogenase [ubiquinone] iron-sulfur subunit, mitochondrial
XPO1	1	A0A7I2V2S3	Exportin-1
DNAJB1	1	M0R080	DnaJ homolog subfamily B member 1
ECHS1	1	A0A384MDW7	Enoyl Coenzyme A hydratase, short chain, 1, mitochondrial

Supplementary Table 8. Summary of mass spectrometry (MS) analysis of WSB2 interactors in HepG2

Identified proteins	Unique Peptides	Protein Accession	Protein Description
WSB2	10	F5H280	WD repeat and SOCS box-containing protein 2
KCTD2	2	B3KT06	BTB/POZ domain-containing protein KCTD2
ERLIN1	1	O75477	Erlin-1
HNRNPA3	2	P51991	Heterogeneous nuclear ribonucleoprotein A3
KCTD5	2	Q9NXV2	BTB/POZ domain-containing protein KCTD5
RPS23	3	P62266	40S ribosomal protein S23
RPL9	2	A0A2R8Y5Y7	60S ribosomal protein L9
PPM1B	3	Q4J6C0	PPM1B beta isoform variant 6
CNFN	1	Q9BYD5	Cornifelin
COL1A2	2	A0A384MDU2	Collagen, type I, alpha 2, isoform CRA_c
POLDIP3	2	Q9BY77	Polymerase delta-interacting protein 3
FLNB	2	A0A024R321	Filamin B, beta (Actin binding protein 278), isoform CRA_a
HRMT1L2	2	Q5U8W9	Protein arginine methyltransferase 1 isoform 4
SERPINB13	2	Q9UIV8	Serpin B13
ZC3HAV1	2	Q7Z2W4	Zinc finger CCCH-type antiviral protein 1
IMPA2	1	K7EL65	Inositol monophosphatase 2
ACSL4	1	A0A804HI36	Long-chain-fatty-acid--CoA ligase 4
MRM3	2	Q9HC36	rRNA methyltransferase 3, mitochondrial
FABP3	2	P05413	Fatty acid-binding protein, heart

TP53	2	L0EQ92	Tumor suppressor p53
ANXA3	2	D6RA82	Annexin
AK4	2	P27144	Adenylate kinase 4, mitochondrial
APOA2	2	V9GYM3	Apolipoprotein A-II
SF3B2	2	H0YCG1	Splicing factor 3B subunit 2
NDUFV1	2	G3V0I5	NADH dehydrogenase [ubiquinone] flavoprotein 1, mitochondrial
JCHAIN	1	C9JA05	Immunoglobulin J chain
RPL37A	1	P61513	60S ribosomal protein L37a
MGME1	1	Q5QPE8	Mitochondrial genome maintenance exonuclease 1
SARS2	1	M0QWZ7	Seryl-tRNA synthetase
MRPS22	2	H7C5L9	28S ribosomal protein S22, mitochondrial
RHOT1	1	H7BXZ6	Mitochondrial Rho GTPase
RSL1D1	1	I3L3C4	Ribosomal L1 domain-containing protein 1
GTPBP6	1	O43824	Putative GTP-binding protein 6
NDUFS7	1	F5H5N1	Complex I-20kD
DNAJB11	2	Q9UBS4	DnaJ homolog subfamily B member 11
ABCF1	1	Q2L6I2	ABC50 protein
MRPS7	1	J3QKW2	28S ribosomal protein S7, mitochondrial
NDUFB10	1	H3BPJ9	Complex I-PDSW
SF3B3	1	Q15393	Splicing factor 3B subunit 3
TRIM21	1	P19474	E3 ubiquitin-protein ligase TRIM21
ARCN1	1	P48444	Coatomer subunit delta
TIMP3	1	P35625	Metalloproteinase inhibitor 3
MRPS28	1	H7C5V3	28S ribosomal protein S28, mitochondrial
NEU2	1	Q9Y3R4	Sialidase-2

CPE	1	C9JE88	Carboxypeptidase E
ELOB	1	Q15370	Elongin-B
ELA2	1	B2MUD5	Neutrophil elastase
CTSB	1	R4GMQ5	Cathepsin B
BAG6	1	F6TC96	Large proline-rich protein BAG6
MRPL16	1	E9PI14	39S ribosomal protein L16, mitochondrial
ATP6	1	A0A343R0H4	ATP synthase subunit a
THEM6	1	Q8WUY1	Protein THEM6
TIMM44	1	O43615	Mitochondrial import inner membrane translocase subunit TIM44
CKM	1	P06732	Creatine kinase M-type

Supplementary Table 9. Primers for qPCR

Primer name	Primer sequence 5’-3’
WSB2_F	GTTAATTCGGAAGCTAGAGG
WSB2_R	CAAAGCCCATTGGTCATA
β-actin_F	GTGAAGGTGACAGCAGTCGGTT
β-actin_R	GAAGTGGGGTGGTTTTAGGA
PTEN_F	CTCAGCCGTTACCTGTGTGT
PTEN_R	AGGTTTCCTCTGGTCCTGGT
TSC2_F	TACGAGTGCAACCTGGTGTC
TSC2_R	GAGGCCATATTTGCGTGCAG
AMPK1_F	AAAGTCGGCGTCTGTTCCAA
AMPK1_R	GGGCCTGCATACAATCTTCCT
IGFBP3_F	TGTGGCCATGACTGAGGAAA
IGFBP3_R	TGCCGACCTTCTTGGGTTT
PHLDA3_F	CAGTAGGGGCTGAGCATGAA
PHLDA3_R	GCAGTCTGCAGAACCCAGAA
TP53_F	CAGCACATGACGGAGGTTGT
TP53_R	TCATCCAAATACTCCACACGC
FAS_F	ATTCTGCCATAAGCCCTGTC
FAS_R	TGGTGTTGCTGGTGAGTGTG
BAX_F	GACAGGGACATCAGTCGCTT

BAX_R	AGACCGTGACCATCTTTGTG
TIGAR_F	CAGCCAGTGTCTTAGTTGTG
TIGAR_R	AACTTCTCTTCCTTCCTCAA
GLS2_F	AGGAAGGAATCCCCCATAAC
GLS2_R	CAAGTGACCTCCACAGAACA
PRKAB1_F	AATCAAGGCACCAGAGAAGG
PRKAB1_R	CCAGTTGTTGAAGGACCCAG
DRAM1_F	CGCCTTCATTATCTCCTACG
DRAM1_R	CCATTCCGAAACATCCCACC
POLH_F	CTTGCTACTCGGGAACAGGT
POLH_R	TGGCAGAGAACTTGGGTCAC
PCNA_F	CTATGAAATGAAGTTGATGG
PCNA_R	AGTGTCACCGTTGAAGAGAG
CDKN1A_F	CCACTGGAGGGTGACTTCGC
CDKN1A_R	CCTGCCTCCTCCCAACTCAT
BTG2_F	AGGCACTCACAGAGCACTAC
BTG2_R	TCTTGTGGTTGATGCGAATG

Supplementary Table 10. Sequences for siRNAs

Gene (siRNA)	Sense 5’-3’	Antisense 5’-3’
WSB2#1	CAUCCCUAAAGGGUUUGAA	UUCAAACCCUUUAGGGAUG
WSB2#2	CGGCUUCUUACGAUACCAA	UUGGUAUCGUAAGAAGCCG
WSB2#3	GACACUGCAUCGUCAAACU	AGUUUGACGAUGCAGUGUC
Sicontrol	UUCUCCGAACGUGUCACGU	ACGUGACACGUUCGGAGAA

Supplementary Table 11. Antibodies and dilutions used in this study

Antibody	Dilutions for WB	Dilutions for IHC	Dilutions for IF	Dilutions for IP	Company
Rabbit anti-WSB2	1:1000	1:100	1:50	1:50	Proteintech
Mouse anti-p53	1:1000	1:100	1:100	1:40	Santa Cruz
Rabbit anti-p53	1:1000	/	/	1:100	Proteintech
Mouse anti-β-actin	1:20000	/	/	/	Proteintech
Mouse anti-Flag	1:1000	/	/	1:200	Sigma-Baldric
Rabbit anti-Flag	1:1000	/	/	1:200	Proteintech
Rat anti-HA	1:1000	/	/	1:100	Roche
Rabbit anti-HA	1:1000	/	/	1:100	Proteintech
Goat anti-mouse IgG HRP	1:5000	/	/	/	CST
Goat anti-rabbit IgG HRP	1:5000	/	/	/	CST
Goat anti-rat IgG HRP	1:5000	/	/	/	CST
Goat Anti-Rabbit IgG (alexa fluor 594)	/	/	1:200	/	Invitrogen
Goat Anti-Mouse IgG (alexa fluor 488)	/	/	1:200	/	Invitrogen

HRP- Mouse Anti-Rabbit IgG Light chain specific	1:2000	/	/	/	Proteintech
Rabbit anti-mTOR	1:1000	/	/	/	CST
Rabbit anti-p-mTOR (Ser2448)	1:1000	1:100	/	/	CST
Rabbit anti-p70S6K	1:1000	/	/	/	CST
Rabbit anti-p-p70S6K (Thr389)	1:1000	/	/	/	CST
Rabbit anti-4E-BP1	1:1000	/	/	/	CST
Rabbit anti-p-4E-BP1 (Thr37/46)	1:1000	/	/	/	CST
Rabbit anti-IGFBP3	1:1000	/	/	/	Proteintech
Rabbit anti-AKT	1:1000	/	/	/	CST
Rabbit anti-p-AKT (Ser473)	1:1000	/	/	/	CST
Mouse anti-Mdm2	1:1000	/	/	/	Santa Cruz
